# Super-enhancers mediates SLC7A11 via FOXA1 to regulate disulfidptosis in prostate cancer

**DOI:** 10.1038/s41419-025-08227-2

**Published:** 2025-12-03

**Authors:** Zhen Kang, Bin Lin, Zhi-Bin Ke, Qing-Shui Zheng, Xue-Yi Xue, Yong Wei, Ning Xu

**Affiliations:** 1 Department of Urology, Urology Research Institute, The First Affiliated Hospital, Fujian Medical University, Fuzhou, China; 2 Department of Urology, National Region Medical Centre, Binhai Campus of The First Affiliated Hospital, Fujian Medical University, Fuzhou, China; 3 Fujian Key Laboratory of Precision Medicine for Cancer, The First Affiliated Hospital, Fujian Medical University, Fuzhou, China

**Keywords:** Prostate cancer, Cell death, Cytoskeleton

## Abstract

Prostate cancer (PCa) remains a major therapeutic challenge due to aberrant androgen receptor signaling and a remodeled tumor microenvironment. Disulfidptosis, a recently identified form of cell death characterized by cytoskeletal collapse under conditions of glucose deprivation and elevated SLC7A11 expression, presents a potential novel avenue for intervention. In this study, we integrated TCGA and GEO data and employed machine learning techniques to identify disulfidptosis-related genes in prostate cancer. Functional analyses using SLC7A11-overexpressing and knockout cell lines demonstrated that SLC7A11 promotes cellular proliferation, migration, and invasion, while its overexpression under glucose-starved conditions triggers disulfidptosis, also inducible pharmacologically using the glucose uptake inhibitor BAY-876. Through CUT&Tag, ChIP-seq, and luciferase assays, we identified FOXA1 as a key transcriptional regulator of SLC7A11, driven by a super-enhancer located at chr14:37583488–37589585. CRISPR-Cas9 deletion of this super-enhancer reduced FOXA1 and SLC7A11 expression, thereby protecting cells from disulfidptosis. These findings highlight the critical role of the SE/FOXA1/SLC7A11 regulatory axis in driving both disulfidptosis and tumor progression, suggesting that targeting this pathway, particularly in glucose-deprived tumor environments, may offer promising therapeutic strategies for PCa.

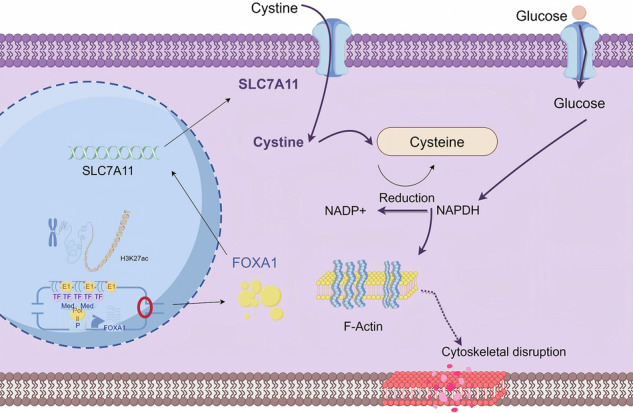

## Introduction

Prostate cancer (PCa) is one of the most common malignant tumors of the male genitourinary system, accounting for approximately 7.3% of all cancer cases worldwide. In Western countries, PCa represents about 20% of newly diagnosed cancers in men, with a mortality rate of around 9%. Although the incidence of PCa is relatively lower in Asian populations, it has been steadily rising in China due to lifestyle changes and an aging population [1, 2]. Early-stage PCa typically responds well to androgen deprivation therapy (ADT); however, most advanced cases eventually progress to castration-resistant prostate cancer (CRPC), which relies more heavily on the androgen receptor (AR) signaling pathway [3]. As a so-called “immune cold” tumor, PCa responds poorly to immune checkpoint inhibitors [4], making the treatment of CRPC particularly challenging.

Programmed cell death is a critical mechanism for eliminating abnormal cells and includes various subtypes such as ferroptosis [5], autophagy, immunogenic cell death [6], and necroptosis [7], all of which have shown emerging potential in cancer therapy. Studies have demonstrated that ferroptosis [8], autophagy, and lysosome-dependent cell death [9] exhibit tumor-suppressive functions across multiple cancer models. Recently, a novel form of programmed cell death termed disulfidptosis has been proposed, driven by dependent on cellular uptake and metabolism of cystine [10]. Tumor cells with high SLC7A11 expression are unable to effectively reduce cystine under glucose-limiting conditions, resulting in aberrant disulfide bond accumulation within the actin cytoskeleton, causing cytoskeletal collapse and subsequent cell death. SLC7A11 is overexpressed in several cancers [11–13], including PCa, and may play a key role in disulfidptosis regulation [14, 15]. Interfering with SLC7A11 expression, in combination with glucose metabolism inhibitors, has been shown to enhance PCa cell sensitivity to disulfidptosis and suppress proliferation. However, the regulatory mechanisms underlying disulfidptosis in prostate cancer remain poorly understood, and upstream transcriptional regulators have yet to be clearly identified.

Super-enhancers (SEs) are cis-regulatory elements with strong transcriptional activity. Enriched with epigenetic markers such as H3K27ac, SEs are closely associated with the expression of critical oncogenes in various cancers [16, 17]. SEs can recruit large numbers of transcription factors and coactivator proteins (e.g., BRD4, MED1) to drive the transcription of oncogenes such as MYC [18], making them important therapeutic targets. In PCa, AR signaling also regulates key oncogenic drivers through SEs [19].

This study aims to investigate the functional role of SE-mediated epigenetic regulation of SLC7A11 and its associated pathways in disulfidptosis within prostate cancer, by integrating pharmacological intervention, gene editing, and high-throughput omics approaches. The goal is to elucidate the transcriptional regulatory mechanisms of disulfidptosis in PCa cells and provide a theoretical foundation for the development of novel targeted therapies.

## Materials and methods

### Bioinformatics analysis

Transcriptomic and clinical data were obtained from public databases TCGA-PRAD (2022 update), GSE70770, and GSE21032. In TCGA-PRAD, “biochemical recurrence” was defined as disease progression, and “days to first biochemical recurrence” was used as the time-to-event variable; for cases without recurrence, “days to last follow-up” was used. In GSE70770, “biochemical relapse” and “time to BCR” were used, while “Disease Free Status” served as the clinical endpoint in GSE21032. After batch correction and integration of the two GEO datasets (GSE70770 and GSE21032) using the sva package, these were combined with the independently used TCGA-PRAD cohort, resulting in a total of 837 PCa samples included in the study. Associations between disulfidptosis-related genes and disease progression were analyzed using the survival and limma packages. To identify the most robust predictive model, 10 algorithms (including RSF, Enet, Lasso, Ridge, CoxBoost, SuperPC) and 111 model combinations were assessed. Leave-one-out cross-validation was conducted in the TCGA cohort, and models were validated in GSE70770 and GSE21032. The model with the highest mean C-index was selected as optimal (Supplementary File 1). Gene co-expression analysis was performed using the GEPIA2 [20] web tool ([http://gepia2.cancer-pku.cn/]) based on TCGA Prostate Adenocarcinoma (PRAD) tumor samples. Pearson correlation coefficients (R) were calculated to assess pairwise gene expression associations. Statistical significance (p-values) was automatically adjusted for multiple testing by GEPIA2 using the Benjamini-Hochberg (BH) method to control the false discovery rate (FDR), and results are presented with FDR-adjusted p-values (adj.P.Val). Significant correlations were defined as those with |R | > 0.3 and adj.P.Val < 0.05.

### Immunohistochemistry (IHC)

Twelve post-surgical PCa tissue specimens from Fujian Medical University Union Hospital, with written informed consent obtained from all patients prior to specimen collection, were fixed in formalin, dehydrated, embedded in paraffin, and sectioned. Following antigen retrieval and blocking, sections were incubated with primary and secondary antibodies against SLC7A11. DAB was used for chromogenic detection and hematoxylin counterstained. Stained slides were examined under a light microscope, and staining intensity and proportion of positive cells were scored. This study was approved by the Ethics Committee of the First Affiliated Hospital of Fujian Medical University (No. MRCTA, ECFAH of FMU [2022]432).

### Cell lines and culture

Human prostate cancer (PCa) cell lines (PC-3, DU145, 22RV1, LNCaP, and C4-2), prostate epithelial cells (RWPE-1), benign prostatic hyperplasia cells (BPH-1), and HEK293T cells were obtained from the Cell Bank of the Chinese Academy of Sciences (Shanghai, China). All cell lines were maintained in Roswell Park Memorial Institute-1640 (RPMI-1640) medium (Gibco), supplemented with 10% fetal bovine serum (FBS, Gibco) and 1% penicillin-streptomycin (Gibco). Cells were incubated at 37 °C in a humidified atmosphere containing 5% CO₂.

### Reagents

SLC7A11 antibody (PROTEINTECH GROUP, INC, 26864-1-AP), FOXA1 antibody (Immunoway, YT5324), glucose-free RPMI-1640 medium (MeilunBio, MA0555), Annexin V-FITC/7-AAD Apoptosis Detection Kit (Yeasen Biotechnology, 40311ES20), Cysteine Uptake Fluorometric Assay Kit (Elabscience, E-BC-F066), NADP⁺/NADPH Colorimetric Assay Kit (Elabscience, E-BC-K803-M), Glutathione (GSH) ELISA Kit (Elabscience, E-EL-0026), Blue/Clear Native PAGE Kit (MeilunBio, MA0472), G6PDH Activity Assay Kit (Beyotime, S0189), G6P Activity Assay Kit (Beyotime, S0185), I-BET151 (ApexBio, B1500), JQ-1 (ApexBio, A1910), and BAY-876 (MedChemExpress, HY-100017).

### Western blotting

Total cellular protein was extracted and quantified using a BCA assay. Equal amounts of protein were denatured, separated by SDS-PAGE, and transferred to PVDF membranes. Membranes were blocked with non-fat milk, then incubated with primary antibody against SLC7A11 and HRP-conjugated secondary antibody. Protein bands were visualized using ECL and quantified with Image J.

### qRT-PCR

Total RNA was extracted using TRIzol or commercial kits, with RNase inhibitors added to prevent degradation. RNA purity was confirmed by spectrophotometry (A260/A280 = 1.8–2.0). Reverse transcription was performed using random hexamers or oligo(dT) primers. Gene expression was quantified via SYBR Green or TaqMan-based qPCR. CT values were analyzed using the 2⁻ΔΔCt method to assess relative gene expression (Supplementary File 1).

### Cell migration and invasion assays

To assess the effects of SLC7A11 knockdown and overexpression on cell migration and invasion, two complementary assays were performed: Transwell and wound healing. For the Transwell assay, cells were serum-starved for 36 h and then seeded into the upper chambers of Transwell inserts (pre-coated with Matrigel for invasion assays). The lower chambers were filled with medium containing 30% FBS as a chemoattractant. After 48 h of incubation, migrated or invaded cells on the lower surface of the membrane were fixed, stained, and counted under a microscope. Image J software was used for quantitative analysis. For the wound healing assay, SLC7A11-silenced and overexpressed cells were seeded in 6-well plates (1 × 10^6^ cells/well) and grown to ~90% confluence. After removing the culture medium and rinsing with PBS, serum-free medium was added. A sterile 20 μL pipette tip was used to make a cross scratch in each well. Cell migration into the wound area was monitored at 0 and 24 h using microscopy. Wound closure was quantified as the change in wound width.

### Flow cytometry

DU145 and PC-3 cells were cultured in glucose-containing or glucose-free media for 24 h, digested, washed with cold PBS, and resuspended at 1 × 10⁶–10⁷ cells/mL. Apoptosis was detected using an Annexin V-FITC/7-AAD kit, followed by incubation in the dark for 15–20 min. At least 10,000 events were acquired on a flow cytometer and analyzed using FlowJo to determine early and late apoptotic populations.

### Immunofluorescence

DU145 and PC-3 cells were cultured on coverslips in 24-well plates, fixed with 4% paraformaldehyde, permeabilized with 0.1% Triton X-100, and blocked with 5% BSA or 10% normal goat serum. Cells were incubated with phalloidin (for actin cytoskeleton) and counterstained with DAPI. Images were acquired using a fluorescence microscope.

### Xenograft Model

PC-3 and DU145 cells (3 × 10⁷/100 μL) were mixed with matrix gel (1:1:3 ratio of concentrated gel: diluted gel: medium) and subcutaneously injected into the axilla of nude mice (*n* = 5/group: SLC7A11-OE, sgRNA#1, sgRNA#2, NC). Tumor volume was measured every 3 days starting from day 6. Mice were sacrificed on day 30 and tumors were weighed.

### ChIP-seq analysis

ChIP-seq analysis of H3K27ac was conducted using datasets from GEO: PC-3 (GSE124769), 22Rv1 (GSE105627), LNCaP (GSE73783), C4-2B (GSE105424), and RWPE-1 (GSE105290). Raw FASTQ files, including IP and input controls, were quality-checked with FastQC and MultiQC (Q scores > 30), and trimmed with Trimmomatic when necessary. Clean reads were aligned to the GRCh38 genome using Bowtie2, and PCR duplicates were removed with Samtools and Picard. DeepTools normalized normalize signals into RPKM. Peaks were called with MACS2 (*p* < 1e-5), and super-enhancers were identified using ROSE by ranking H3K27ac signal intensities to detecting the inflection point. Super-enhancer regions were annotated with nearby genes based on genome annotation. VennDiagram visualized overlaps of super-enhancers and target genes across cell lines. Functional enrichment of target genes was performed using HOMER or GREAT, and IGV was used to visualize H3K27ac signal distribution.

### CUT&Tag sequencing

Cells were fixed with 0.1% formaldehyde for 10 min, and crosslinking was quenched with 125 mM glycine. After washing, nuclei were released and incubated with magnetic beads modified for nuclear binding. H3K27ac-specific primary antibodies were added and incubated at 4 °C for 2 h, followed by pA-Tn5 transposase-conjugated secondary antibodies for 1 h. Transposition was activated with Mg²⁺ and carried out at 37 °C for 1 h to cleave H3K27ac-associated chromatin. DNA fragments were purified and used for library preparation with a high-throughput kit, followed by Illumina sequencing (150 × 150 paired-end sequencing on the Illumina NovaSeq 6000 instrument).

FastQC was used for quality control, and Bowtie2 aligned clean reads to the GRCh38 (hg38) genome. Low-quality and multi-mapped reads were filtered to ensure high-confidence data for downstream analysis. Enriched regions (peaks) of H3K27ac were identified using MACS2 (v2.1.2) with parameters -m 5 50. These peaks were used as constituent enhancers for super-enhancer (SE) identification via ROSE software (https://bitbucket.org/young_computation/rose). For ROSE analysis, the key parameters were set as follows: a stitching distance of 12,500 bp (i.e., constituent enhancers within 12,500 bp were stitched into a single region); peaks within ±2500 bp of annotated transcription start sites (TSS) were excluded from stitching to avoid promoter regions. Super-enhancers were distinguished from typical enhancers by the inflection point of H3K27ac signal intensity versus enhancer rank, as determined by the ROSE algorithm. Enhancer annotation was performed using Homer (v4.10.4) utility annotatePeaks.pl to associate peaks with their nearby genes.

### CRISPR-Cas9 knockout

sgRNAs targeting the defined subregions of the FOXA1 super-enhancer (SE-sgRNA-1, SE-sgRNA-2, SE-sgRNA-3) and the core promoter region (Pr-sgRNA) were designed using CRISPOR based on ChIP/CUT&Tag data and validated for specificity by BLAST analysis against the human genome. For each target (three SE subregions and the promoter), four tandem sgRNAs were cloned into the lentiCRISPR v2 vector (Addgene #52961). Correct recombination was confirmed by Sanger sequencing of positive clones, and all sgRNA oligonucleotide sequences are provided in Supplementary File 1. Lentiviruses were produced in 293 T cells by co-transfecting transfer vectors (lentiCRISPR v2 containing sgRNAs), psPAX2, and pMD2.G. Viral supernatants were collected and used to infect DU145 and PC-3 cells. Forty-eight hours post-infection, cells were selected with 2 µg/mL puromycin for 7 days to establish stable knockout pools. The resulting cell groups were: wild-type (WT), individual SE subregion knockouts (SE1-KO, SE2-KO, SE3-KO), promoter knockout (Pr-KO), and combinatorial SE knockout (3xSE-KO). The 3xSE-KO pool was generated by sequentially transducing DU145 and PC-3 cells with lentiviruses expressing SE-sgRNA-1, SE-sgRNA-2, and SE-sgRNA-3, followed by pooled puromycin selection (2 µg/mL, 7 days) after the final infection.

Knockout efficiency was evaluated based on the biological consequence of super-enhancer or promoter functional loss: downregulation of FOXA1 expression. FOXA1 transcript levels were measured by RT-qPCR and protein levels were assessed by Western blot (immunoblotting) in all cell line groups (WT, SE1-KO, SE2-KO, SE3-KO, Pr-KO, 3xSE-KO). To elucidate the relative contribution of each SE sub-region and the combinatorial effect, FOXA1 expression (mRNA and protein) was compared between the following groups: Each individual SE sub-region knockout (SE1-KO, SE2-KO, SE3-KO) vs. WT; The promoter knockout (Pr-KO) vs. WT; The combinatorial SE knockout (3xSE-KO) vs. WT; The combinatorial SE knockout (3xSE-KO) vs. promoter knockout (Pr-KO).

### Targeted metabolomics sequencing

To investigate the regulatory role of SLC7A11 in cellular energy metabolism, DU and PC cells were subjected to CRISPR/Cas9-mediated SLC7A11 knockout (experimental group) with non-knockout cells as controls (nc). After 48 h of culture, cells were harvested and subjected to targeted metabolomics analysis at Meiji Bio, covering 106 metabolites (Supplementary File 1) associated with energy metabolism, including carbohydrate, amino acid, and nucleotide pathways. Metabolites were extracted using pre-cooled methanol–water solution, vortexed, centrifuged at low temperature, and analyzed on a UPLC–MS/MS platform. Data processing was performed using the Meiji Bio analysis platform. Raw data were log₂-transformed, and PCA was applied for quality control. Samples failing QC were excluded from further analysis. Differential metabolites were screened using thresholds of FC > 1 or < 1, *p* < 0.05, and VIP ≥ 1. KEGG pathway enrichment was performed using a hypergeometric distribution algorithm, with p-values adjusted via the Benjamini–Hochberg method. Pathways with adjusted *p* < 0.05 were considered significantly enriched.

### Transcriptome sequencing

Total RNA was extracted from DU145 and PC-3 cells treated with iBET-151. Libraries were constructed and sequenced using Illumina NovaSeq 6000. Raw reads were quality-checked (FastQC), aligned to GRCh38 (HISAT2), and differentially expressed genes were identified with DESeq2 (*p* < 0.05, log2FC > 1). GO/KEGG enrichment and PCA heatmaps were used for downstream analysis.

### Transcription factor binding validation

UCSC and JASPAR databases were used to predict potential transcription factor binding sites in the SLC7A11 promoter. FOXA1 was selected and its structure predicted via AlphaFold3. PyMOL was used for docking simulations. Wild-type and mutant promoter luciferase reporter constructs were transfected into HEK-293T cells to assess FOXA1 transcriptional activity. CUT&Tag-qPCR was used to validate FOXA1 binding in PCa cells.

### In vivo drug intervention

Transfected PC-3 and DU145 cells (5 × 10⁷/100 μL) were injected subcutaneously into nude mice. Once tumors reached ~80 mm³, mice received intraperitoneal injections of BAY-876 (3 mg/kg, 0.1 mL/10 g) every 3 days. Tumor growth and body weight were monitored throughout. Tumors were harvested for subsequent analysis.

### Prediction of FOXA1 transcription factor binding sites and structural analysis via AlphaFold3

Using JASPAR, hTFtarget, and UCSC Genome Browser, we identified FOXA1 as a potential regulator of the SLC7A11 promoter. To investigate its binding mechanism, we predicted the 3D structure of FOXA1 with AlphaFold3, using its amino acid sequence from UniProt. The predicted model was used for molecular docking analysis between FOXA1 and the SLC7A11 promoter using PyMOL. Docking results revealed high-affinity interactions at specific promoter nucleotides, indicating potential regulatory elements. Two key binding sites were selected for site-directed mutagenesis and further validation (Supplementary File 1).

### Dual-luciferase reporter assay validation

HEK-293T cells were used as the experimental model. Cells were cultured in 10 cm dishes until reaching 80–90% confluence. The culture medium was discarded, and cells were washed twice with 2 mL PBS. Then, 2 mL of Trypsin-EDTA solution was added, mixed gently, and incubated at 37 °C for 1–5 min. The trypsin solution was removed, and 2 mL of complete medium was added. Cells were gently pipetted to form a single-cell suspension. After cell counting using a hemocytometer, the suspension was diluted to 1×10⁶ cells/mL. Cells were then seeded in 12-well plates at a density of 5 × 10⁵ cells/well and incubated at 37 °C in 5% CO₂ for 24 h. pcDNA3.1( + )-TF (FOXA1) plasmid was dissolved in DEPC water at a final concentration of approximately 20 μM (125 μL per 1 OD). The control group used pcDNA3.1(+) vector in the same manner. For transfection, 100 μL of serum-free medium was added to a 1.5 mL EP tube along with 5 μL of pcDNA3.1( + )-TF and 1 μg of dual-luciferase reporter plasmid, mixed thoroughly. In another EP tube, 100 μL of serum-free DMEM and 4 μL of Lipo8000 were added and mixed, incubated at room temperature for 5 minutes, then combined with the first tube, and incubated for 20 minutes at room temperature. The validation included the following eight experimental groups: A. pcDNA3.1( + )-TF + PGL3-WT.B. pcDNA3.1( + )-TF + PGL3-Mut1 .C. pcDNA3.1( + )-TF + PGL3-Mut2;D. pcDNA3.1(+) + PGL3-WT;E. pcDNA3.1(+) + PGL3-Mut1;F. pcDNA3.1(+) + PGL3-Mut2;G. pcDNA3.1( + )-TF + PGL3-Basic;H. pcDNA3.1(+) + PGL3-Basic. Each group was set up in triplicate. When cells in the 12-well plate reached approximately 70% confluency, the culture medium was removed and the transfection mixture was added dropwise to each well. After gentle mixing, plates were incubated at 37 °C in 5% CO₂ for 24 h. The transfection mixture was then replaced with 500 μL of complete medium and cells were cultured for another 24 or 48 h before sampling. For the dual-luciferase assay, the culture medium was removed and cells were washed twice with 500 μL PBS (carefully to avoid disturbing adherent cells). Then, 300 μL of 1× Passive Lysis Buffer (PLB) was added to each well. The plates were gently agitated at room temperature for 15 minutes to lyse the cells. The lysates were transferred to assay plates (100 μL/well), and each sample was measured in triplicate. After preheating the microplate reader and selecting the dual-luciferase detection protocol, 10 μL of LARII reagent was added per well. After a 1–2 s delay and 5–10 s of reading, firefly luciferase activity was recorded. Then, 10 μL of Stop & Glo® reagent was added to each well, and Renilla luciferase activity was measured under the same conditions.

### CUT&Tag PCR detection

CUT&Tag combined with qPCR was used to assess FOXA1 binding to the SLC7A11 promoter. Cell lines with high FOXA1 expression were cultured to the logarithmic phase and fixed with 1% formaldehyde preserving protein-DNA interactions. Nuclei were isolated and incubated overnight with a FOXA1-specific antibody. Magnetic beads were used to capture antibody-bound complexes, enriching FOXA1-associated DNA. Tagmentation was performed with tagmentase, simultaneously cleaving and tagging FOXA1-bound DNA regions. After pre-amplification and DNA purification, the enriched DNA served as template for qPCR to evaluate binding at the SLC7A11 promoter (Supplementary File 1).

### Statistical analysis

Data were analyzed using SPSS 26.0 software (IBM Corp., Armonk, NY, USA), and graphs were generated with GraphPad Prism 8.0. For normally distributed data, results were expressed as mean ± standard deviation (SD). Comparisons between two groups were performed using the Student’s t-test, while one-way ANOVA followed by post hoc pairwise comparisons was used for comparisons among three or more groups. Primer and plasmid sequences have been added to Supplementary File 1. *P* value < 0.05 was considered statistically significant. Statistical significance was indicated as follows: *P < 0.05, **P < 0.01, ***P < 0.001.

## Results

### SLC7A11 as a biomarker for prostate cancer

**Fig. 1:**
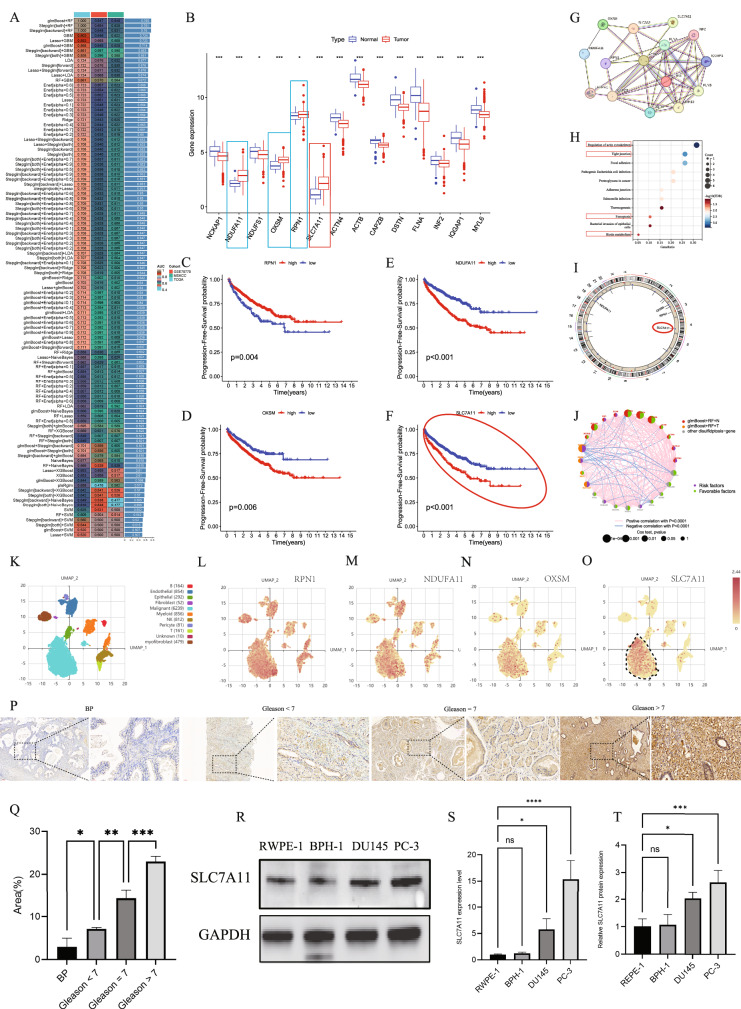
Analysis of biomarkers in prostate cancer. **A** Concordance index (C-index) values of multiple machine learning models across different datasets. **B** Differential expression of ferroptosis-related genes between prostate cancer tissues and normal samples. **C**–**F** Kaplan–Meier curves comparing progression-free survival between high and low expression groups of RPN1, OXSM, NDUFA11, and SLC7A11. **G** Protein–protein interaction network of 15 key ferroptosis-related proteins. **H** Bubble plot of KEGG pathway enrichment for the selected gene set. **I** Chromosomal locations of ferroptosis-related genes in the human genome. **J** Correlation network of ferroptosis-related genes before and after machine learning selection; edges represent correlations between risk and protective factors, with color and thickness indicating correlation strength. **K** UMAP dimensionality reduction analysis of the GSE141445 dataset showing spatial clustering of different tumor cell subtypes. **L**–**O** Expression patterns of RPN1, OXSM, NDUFA11, and SLC7A11 across various tumor cell clusters. **P** Immunohistochemical staining of SLC7A11 in benign prostate (BP) tissues and prostate cancer tissues with different Gleason scores (<7, =7, >7). **Q** Quantitative analysis of SLC7A11-positive areas in immunohistochemistry; higher Gleason scores correlate with significantly increased SLC7A11 expression. **R**–**T** Expression levels of SLC7A11 in different prostate cell lines detected by qRT-PCR and Western blot assays.

The TCGA-PRAD dataset was used as the training cohort, while GSE70770 and GSE21032 were merged after batch effect correction and served as the testing cohort (Supplementary Fig. 1A). Based on 24 disulfidptosis-related genes, a prostate cancer diagnostic model was constructed using 113 algorithmic combinations. Among these, the glmBoost + RF model achieved the highest concordance index (C-index) of 0.76 (Fig. 1A). This model included 15 genes such as SLC7A11, FLNA, ACTB, and MYH10 (Supplementary File 1). Among them, SLC7A11, OXSM, RPN1, and NDUFA11 showed higher expression levels in prostate cancer tissues compared to normal samples (Fig. 1B). Building on our previous research [15], further analysis of tumor progression within the training set revealed that RPN1 may act as a suppressor of prostate cancer progression (Fig. 1C), while OXSM, NDUFA11, and SLC7A11 appear to promote disease progression (Fig. 1E, F). At the protein level, these model genes demonstrated interactions centered around ACTB (Fig. 1G), suggesting their potential involvement in tumorigenesis and progression. Functional enrichment analysis showed that these genes are mainly associated with pathways such as actin cytoskeleton organization, cell junctions, biotin metabolism, and ferroptosis (Fig. 1H). Given the pivotal role of SLC7A11 in cancer development and its function as a “trigger” in multiple forms of regulated cell death, SLC7A11—located on human chromosome 4—was selected as the primary focus of this study (Fig. 1I). Notably, SLC7A11 showed strong positive correlations with OXSM and NDUFA11 (Fig. 1J).

Single-cell RNA sequencing data from 13 prostate tumor samples in the GSE141445 dataset (Fig. 1K) revealed that RPN1 is expressed across various prostate cancer cell clusters, including NK cells, macrophages, and malignant epithelial cells (Fig. 1L), whereas OXSM and SLC7A11 are predominantly expressed in malignant epithelial cells (Fig. 1M–O), which is consistent with our previous findings [15]. Immunohistochemical analysis indicated that SLC7A11 expression was lowest in benign prostate (BP) samples, moderately elevated in prostate cancer samples with Gleason scores <7, and significantly increased in tumors with Gleason scores of 7 and >7, with the highest levels observed in the >7 group (Fig. 1P). Quantitative analysis further confirmed that SLC7A11 expression was significantly higher in tumor tissues compared to normal tissues, and its expression levels increased with higher Gleason scores (Fig. 1Q). In vitro experiments using qRT-PCR and Western blotting demonstrated the highest SLC7A11 expression in the PC-3 cell line, slightly lower levels in DU145, and minimal expression in BPH-1 and RWPE-1 (normal prostate epithelial cells) (Fig. 1R–T). These findings suggest that elevated SLC7A11 expression may be closely associated with the onset and progression of prostate cancer, highlighting its potential as a novel biomarker.

### SLC7A11 promotes apoptosis under glucose-deprived conditions

**Fig. 2:**
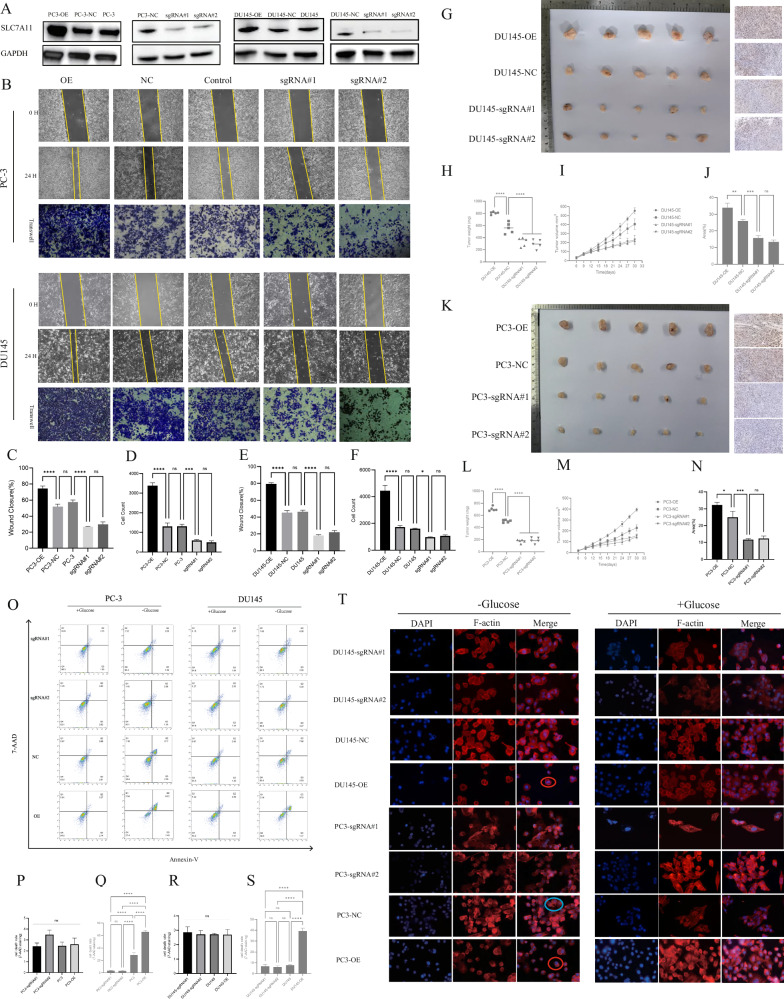
Effects of SLC7A11 on prostate cancer cell viability. **A** Western blot showing SLC7A11 expression in different treatment groups. **B**–**F** Wound healing and Transwell assays showing enhanced migration/invasion in SLC7A11-overexpressing (OE) cells and reduced abilities in knockout cells (sgRNA#1, sgRNA#2). **G** Tumor morphology after subcutaneous injection of DU145 cells (OE, NC, sgRNA#1, sgRNA#2) in nude mice. **H** Tumor weight comparison. **I** Tumor growth curves. **J** Ki67 immunohistochemistry in DU145 tumors. **K** Tumor morphology after PC-3 cell injection (OE, NC, sgRNA#1, sgRNA#2). **L** Tumor weight comparison. **M** Tumor growth curves for PC-3 xenografts. **N** Ki67 immunohistochemistry in PC-3 tumors. **O** Annexin V/7-AAD flow cytometry plots of PC-3 and DU145 cells under glucose-sufficient (+Glucose) and glucose-deprived (−Glucose) conditions. **P**–**S** Apoptosis rate statistics for PC-3 and DU145 cells. **T** F-actin staining in DU145 and PC-3 cells under +Glucose and −Glucose conditions.

Firstly, we evaluated the in vitro effects of SLC7A11 on the migratory capabilities and viability of prostate cancer cells. Western blot confirmed successful overexpression and knockout of SLC7A11 in DU145 and PC-3 cell lines via lentiviral and CRISPR transduction, with protein levels showing significant differences compared to negative control (NC) and wild-type (WT) groups (Fig. 2A). Wound healing and Transwell assays demonstrated that SLC7A11 overexpression significantly enhanced cell migration and invasion in both DU145 and PC-3 cells, whereas knockout markedly reduced these capabilities, indicating a pivotal role of SLC7A11 in promoting the invasive and migratory behavior of CRPC cell lines (Fig. 2B–F). In a xenograft mouse model, subcutaneous injection of DU145 cells revealed that tumors derived from the SLC7A11-overexpressing group (DU145-OE) exhibited significantly greater mass compared to both the NC and knockout groups (DU145-sgRNA#1 and DU145-sgRNA#2) (Fig. 2G). Notably, tumors in the DU145-NC group were also significantly larger than those in the sgRNA groups (Fig. 2H). After 18 days of implantation, tumor volume in the OE group was significantly higher than that in the sgRNA groups, with a similar pattern observed at day 21 in the NC group (Fig. 2I). Immunohistochemical analysis showed that Ki67 expression was highest in the DU145-OE tumor tissues, indicating enhanced proliferative activity (Fig. 2J). In the PC-3 xenograft model, tumors derived from the PC3-OE group also exhibited significantly larger masses (Fig. 2K), whereas tumor weights in the sgRNA groups were markedly reduced compared to both PC3-NC and PC3-OE groups (Fig. 2L). Tumor volume in the OE group was significantly larger on day 15, and by day 24, the NC group also showed significantly larger tumors than the sgRNA group (Fig. 2M). Ki67 expression in the PC3-OE group was substantially higher than in the sgRNA and NC groups (Fig. 2N). These in vivo findings support the critical role of SLC7A11 in promoting prostate cancer growth.

Flow cytometry assessed cell death in SLC7A11-overexpressing and knockout prostate cancer cells (PC-3 and DU145) under different culture conditions (Fig. 2O). In PC-3 cells cultured under glucose-standard conditions (2 g/L), there were no significant differences in late apoptosis among the groups (Fig. 2P). However, under glucose-deprived conditions, the overexpression group exhibited significantly increased apoptosis compared to both the control and knockout groups; the control group also showed higher apoptosis than the knockout group (Fig. 2Q). Similarly, in DU145 cells, no significant differences in apoptosis were observed under glucose-standard conditions (Fig. 2R), but after 24 h of glucose deprivation, the overexpression group displayed significantly higher cell death, with no notable differences between the control and knockout groups (Fig. 2S). These results, together with previous findings [10, 15], suggest that SLC7A11 regulates prostate cancer cell viability without inducing substantial apoptosis under normal conditions. However, under glucose-deprived conditions, high SLC7A11 expression triggers late-stage apoptosis. Under standard culture conditions (+Glucose), DU145 cells exhibited a rounded morphology, while PC-3 cells displayed regular polygonal or spindle-like shapes with well-defined boundaries. F-actin was evenly distributed, forming stable cytoskeletal structures. Overexpression or knockout of SLC7A11 did not affect cytoskeletal morphology. After 24 h of glucose deprivation (-Glucose), DU145-NC cells showed no obvious morphological changes, whereas some PC3-NC cells exhibited cytoskeletal shrinkage (indicated by blue boxes). In SLC7A11-overexpressing groups (DU145-OE and PC3-OE), pronounced F-actin collapse and condensed, rounded morphologies were observed (highlighted in red boxes). Knockout groups displayed no significant changes in cytoskeletal structure (Fig. 2T). These observations indicate that SLC7A11 does not affect cytoskeletal function under normal conditions, but its overexpression under glucose-deprived conditions leads to cytoskeletal collapse in prostate cancer cells.

### iBET-151 suppresses FOXA1 expression

**Fig. 3:**
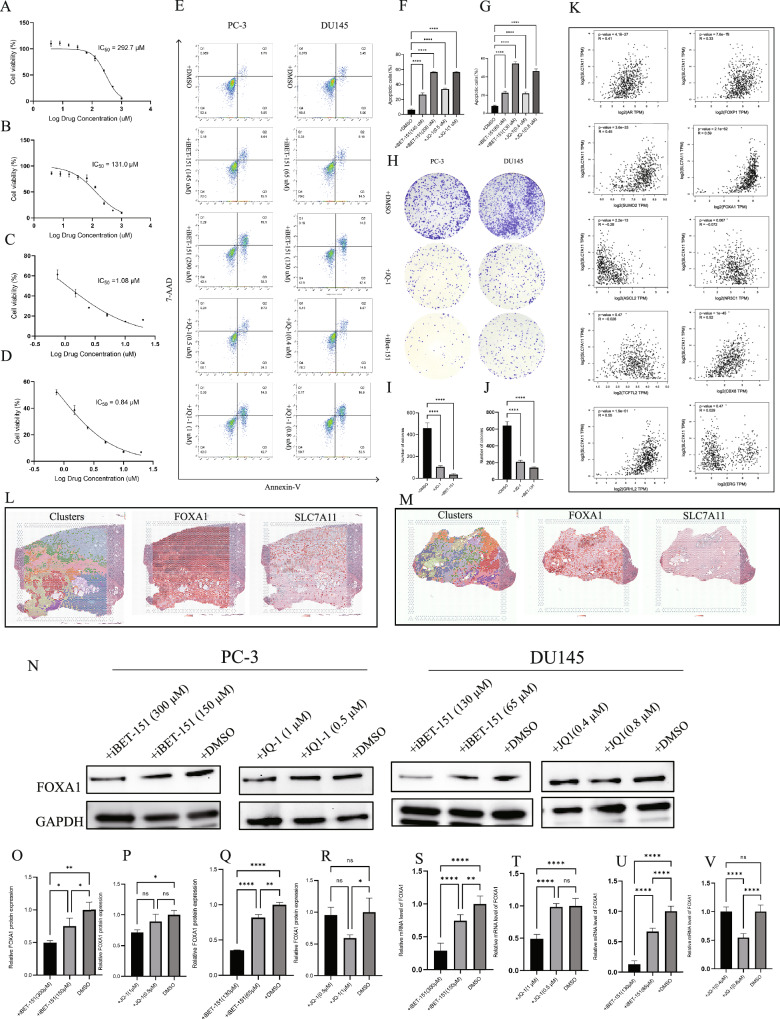
Regulatory effects of iBET-151 on prostate cancer cells. **A**, **B** IC_50_ values of iBET-151 in PC-3 and DU145 cells. **C**, **D** IC_50_ values of JQ-1 in PC-3 and DU145 cells. **E** Flow cytometry analysis of apoptosis in PC-3 and DU145 cells treated with increasing concentrations of iBET-151 and JQ-1. **F**, **G** Quantification of apoptotic cells in PC-3 and DU145 following treatment. **H** Effects of iBET-151 and JQ-1 on colony formation in PC-3 and DU145 cells. **I**, **J** Quantification of colony numbers in treated and control groups. **K** Co-expression analysis of SLC7A11 and 10 predicted transcription factors in prostate tissue, based on TCGA and GTEx transcriptomic datasets. **L** Spatial transcriptomic analysis showing FOXA1 and SLC7A11 expression patterns in prostate cancer tissues. **M** Spatial transcriptomic profiles of FOXA1 and SLC7A11 in normal prostate tissues. **N**–**V** Changes in FOXA1 protein and mRNA levels in PC-3 and DU145 cells treated with iBET-151 (300 μM and 150 μM) and JQ-1 (1 μM and 0.5 μM), as assessed by Western blot and qPCR.

Small molecule inhibitors (iBET-151, JQ-1) can regulate the transcriptional activity of super-enhancer-associated genes by targeting BRD4. The IC50 values of iBET-151 for PC-3 and DU145 cells were 292.7 μM and 131.0 μM, respectively (Fig. 3A, B), while those of JQ-1 were 1.08 μM and 0.84 μM for PC-3 and DU145 cells, respectively (Fig. 3C, D). Compared to the DMSO control group, these inhibitors significantly induced apoptosis in DU145 and PC-3 cells, and the apoptosis rate increased markedly with higher drug concentrations in both iBET-151 and JQ-1 treatment groups (Fig. 3E-G). Furthermore, colony formation assays showed that treatment with iBET-151 (PC3, 145 μM; DU145, 65 μM) and JQ-1 (PC-3, 0.5 μM; DU145, 0.4 μM) significantly reduced the clonogenic capacity of both prostate cancer cell lines (Fig. 3H-J). These findings suggest that iBET-151 and JQ-1 may exert antitumor effects by inducing apoptosis and inhibiting cell proliferation. Using the hTFtarget and JASPAR databases, this study identified 10 potential transcription factors of SLC7A11 in prostate tissue, including FOXA1, ASCL2, GRHL2, AR, TCF7L2, NR3C1, CBX8, FOXP1, SUMO2, and ERG. Based on transcriptome sequencing data from the TCGA and GTEx databases, co-expression analyses were conducted between SLC7A11 and these 10 transcription factors (Fig. 3K). Results showed that except for TCF7L2, NR3C1, and ERG, the remaining 7 transcription factors were significantly correlated with SLC7A11 expression, with FOXA1 showing the highest correlation (*R* = 0.59), suggesting that it may be a key regulator of SLC7A11 in prostate tissue.

After culturing PC-3 (145 μM) and DU145 (65 μM) cells with the SE inhibitor iBET-151 for three days, transcriptome sequencing revealed that among the 10 transcription factors, only FOXA1 was significantly downregulated in the treatment group, while others showed no significant change or were downregulated in controls (Supplementary Fig. 1B, C). Spatial transcriptomic data indicated that FOXA1 and SLC7A11 are highly expressed primarily in tumor epithelial and neuroendocrine cells (Fig. 3L), suggesting that these may be the key cell types through which FOXA1 and SLC7A11 exert their oncogenic roles. In normal prostate-like tissue, both FOXA1 and SLC7A11 showed synchronously reduced expression(Fig. 3M), exhibiting a clear co-expression trend (Supplementary Fig. 1D,E). Western blot results showed that iBET-151 treatment reduced FOXA1 protein levels in PC-3 and DU145 cells (Fig. 3R). At concentrations close to the IC50, FOXA1 protein levels decreased with increasing iBET-151 concentration, falling below 50% of the control group, showing stronger inhibitory effects than JQ-1. In contrast, JQ-1 only partially inhibited FOXA1 expression at concentrations more than twice its IC50. qPCR further confirmed that these drugs suppressed FOXA1 transcriptional activity by reducing its mRNA expression (Fig. 3O-V), with iBET-151 again demonstrating stronger inhibition than JQ-1. This section also analyzed the expression of FOXA1 and SLC7A11 across different prostate cancer cell lines. Results showed that FOXA1 expression was significantly higher in prostate cancer cell lines such as LNCaP, PC-3, and DU145 compared to the normal prostate cell line RWPE-1, and SLC7A11 expression was positively correlated with FOXA1 expression, being notably upregulated in prostate cancer cell lines (Supplementary Fig. 1 F-H).

### Identification of super-enhancers

**Fig. 4:**
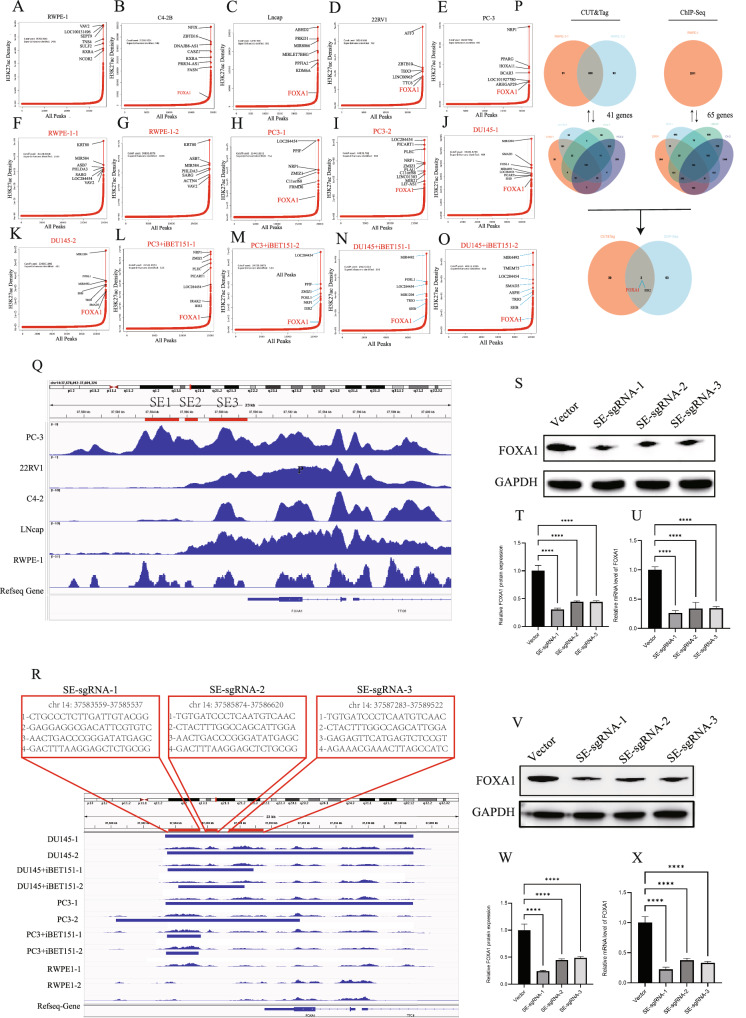
Identification of super-enhancer target genes. **A**–**E** Secondary analysis of ChIP-seq data from the ENCODE public database showing super-enhancer (SE) target genes across various prostate cell lines. **F**–**K** Super-enhancer target genes identified in DU145, PC-3, and RWPE-1 cell lines based on CUT&Tag experimental data. **L**–**O** Changes in SE-associated genes in PC-3 and DU145 cells following iBET-151 treatment. **P** Overlap of SE target genes identified by CUT&Tag and ChIP-seq analyses; FOXA1 and IER2 were the only genes consistently identified. **Q** SE enrichment surrounding the FOXA1 locus across different cell lines based on ENCODE ChIP-seq data. **R** CUT&Tag sequencing results showing SE enrichment at the FOXA1 downstream region; genomic locations and sequences targeted by SE-sgRNA constructs. **S**–**U** In DU145 cells, transfection with SE-sgRNA-1, SE-sgRNA-2, or SE-sgRNA-3 significantly reduced FOXA1 mRNA and protein expression levels. **V**–**X** In PC-3 cells, transfection with SE-sgRNA-1, SE-sgRNA-2, or SE-sgRNA-3 also led to marked downregulation of FOXA1 transcription and protein expression.

In this study, SEs were identified by integrating ChIP-seq and CUT&Tag sequencing with the ROSE algorithm, based on the signal density of H3K27ac modifications across different cell lines. Active enhancers were defined genome-wide according to H3K27ac signal intensity, and the enrichment score for each enhancer was quantified statistically. Enhancers were ranked by enrichment, and the inflection point of the curve with a slope of 1 was used as a threshold to distinguish typical enhancers from super-enhancers. Reanalysis of ChIP-seq data from the ENCODE database revealed significant H3K27ac enrichment across multiple gene regulatory regions in prostate cancer cell lines including PC-3, C4-2B, and 22Rv1. FOXA1 was consistently identified as a super-enhancer target gene across all cancer cell lines, whereas in normal prostate epithelial cells (RWPE-1), FOXA1 was not SE-regulated (Fig. 4A–E). These results suggest that SEs may play a key role in the transcriptional regulation and functional reprogramming of prostate cancer cells. Further analysis of CUT&Tag data focused on H3K27ac signal profiles and SE target gene distribution in DU145, PC-3, and RWPE-1 cells. Compared to RWPE-1, cancer cells showed stronger H3K27ac signals, and MIR1204, IER2, SMAD3, and FOXA1 were SE-associated in PC-3 and DU145 but not RWPE-1 (Fig. 4F–K). After iBET-151 treatment, H3K27ac signals associated with FOXA1 were markedly reduced in both PC-3 and DU145 cells (Fig. 4L–O), supporting FOXA1 as a direct SE target in prostate cancer progression. To further refine SE targets, SE-associated genes were identified in multiple prostate cancer cell lines and intersected with those found in RWPE-1. Differential SE targets were identified by subtracting the RWPE-1 gene set. In total, 41 and 65 differentially enriched genes were identified from CUT&Tag and ChIP-seq data, respectively, with only FOXA1 and IER2 appearing in both datasets (Fig. 4P), highlighting FOXA1 as a conserved, cancer-specific SE target. ChIP-seq analysis of FOXA1 revealed strong H3K27ac signals downstream of the FOXA1 gene, particularly in the PC-3 cell line, where the broadest peak (~10 kb) was observed (Fig. 4Q). In contrast, RWPE-1 exhibited minimal signal in this region, indicating cell line–specific SE activity. As prostate cancer progresses, the regions co-enriched with SEs and FOXA1 expand, suggesting dynamic SE remodeling.

Based on CUT&Tag profiles (Fig. 4R), a potential SE region located at chr14:37583488–37589585 was identified downstream of FOXA1. This region showed strong H3K27ac signals in DU145 and PC-3 cells, which were markedly diminished after 48 hours of iBET-151 treatment, further supporting its identity as a functional SE element regulating FOXA1 transcription. To experimentally validate this region, three CRISPR/Cas9 sgRNAs (SE-sgRNA-1, SE-sgRNA-2, SE-sgRNA-3) were designed to target distinct subregions within the SE locus based on prominent H3K27ac peaks: SE-sgRNA-1: chr14:37583559-37585537; SE-sgRNA-2: chr14:37585874-37586620; SE-sgRNA-3: chr14:37587283-37589522. Each SE-sgRNA construct contained four tandem E-sgRNAs targeting specific segments of the enhancer region (indicated in red boxes in the figures). These constructs were introduced into DU145 and PC-3 cells. Functional assays demonstrated that all three SE-sgRNAs significantly suppressed FOXA1 mRNA and protein levels compared to vector controls, with SE-sgRNA-1 exhibiting the strongest inhibitory effect (Fig. 4S–X). These findings confirm that the downstream SE region is critical for FOXA1 transcriptional regulation. In addition, a luciferase reporter gene assay in HEK-293T was used to validate the ability of SEs to enhance the transcriptional activity of the FOXA1 promoter. First, three segments (300-600 bp) of the FOXA1 promoter region were cloned into the pGL3-basic plasmid (named FOXA1-promoter-1, FOXA1-promoter-2, and FOXA1-promoter-3, respectively) (Supplementary Fig. 1H). The segment with the highest transcriptional activity (FOXA1-promoter-2) was selected for subsequent SE activity analysis (Supplementary Fig. 1I). A negative control site outside the SE region (Control-Luciferase) was used as a control group, while sequences corresponding to the three SE regions were designed as SE-Luciferase constructs and cloned upstream of the FOXA1-promoter-2 vector (Supplementary Fig. 1J). Compared to the Control-Luciferase group, all three SE-Luciferase constructs showed significantly enhanced luciferase activity (Supplementary Fig. 1K).

### Mechanistic investigation of FOXA1 interaction with the SLC7A11 promoter

**Fig. 5:**
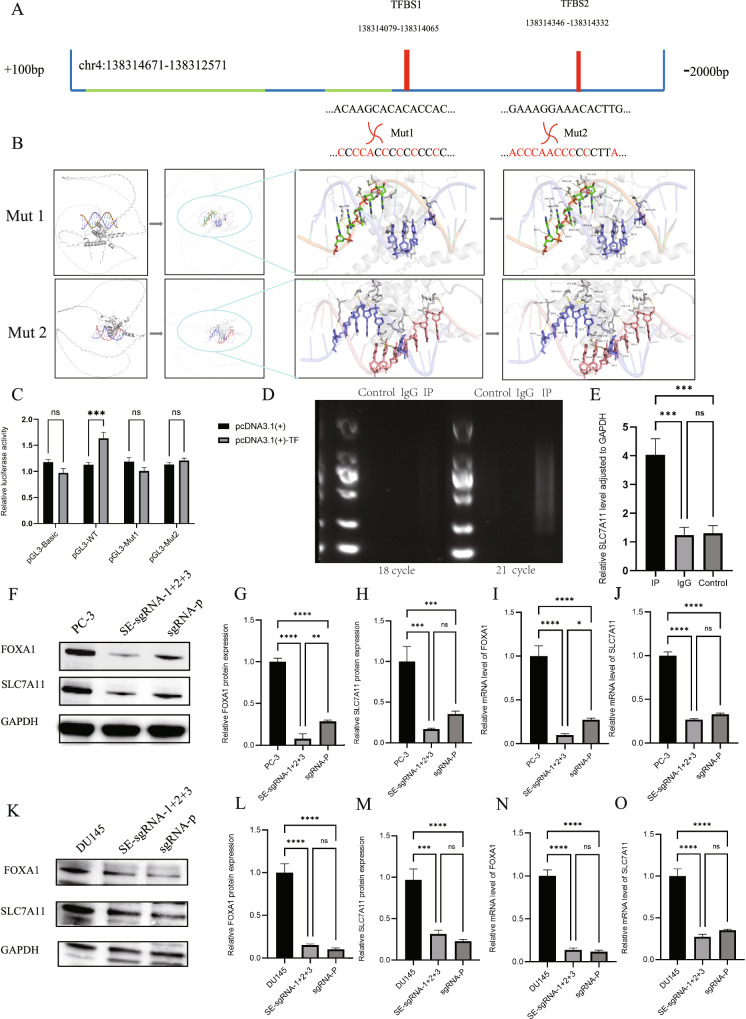
Interaction between FOXA1 and the SLC7A11 promoter and its role in gene regulation. **A** Predicted binding sites of FOXA1 on the SLC7A11 promoter based on AlphaFold-3 modeling. **B** Molecular docking simulation illustrating the interaction between FOXA1 protein and the SLC7A11 promoter region. **C** Dual-luciferase reporter assays show that FOXA1 enhances SLC7A11 promoter activity, while mutation of the binding sites disrupts this regulatory effect. **D** CUT&Tag analysis reveals DNA amplification bands in the FOXA1 immunoprecipitated (IP) group, indicating specific enrichment. **E** qPCR analysis confirms significant enrichment of the SLC7A11 promoter region in the FOXA1-IP group compared to controls. **F**–**J** In PC-3 cells, sequential disruption of the super-enhancer (SE) or promoter region significantly reduces FOXA1 and SLC7A11 expression. **K**–**O** In DU145 cells, similar SE and promoter targeting also leads to a marked downregulation of FOXA1 and SLC7A11, demonstrating consistent regulatory effects.

Candidate binding sites of FOXA1 on the SLC7A11 promoter were screened using the JASPAR database. Two potential transcription factor binding sites (TFBS1 and TFBS2) were identified (Fig. 5A), and site-directed mutagenesis was performed to construct luciferase reporter plasmids carrying mutations at these loci. Using the predicted 3D structure of FOXA1 protein and the sequence of the SLC7A11 promoter region, AlphaFold-3 was employed to model protein–DNA docking interactions. The results visually demonstrated the binding conformation between FOXA1 and the SLC7A11 promoter (Fig. 5B). A dual-luciferase reporter assay was conducted in HEK293T cells to evaluate the transcriptional regulatory function of FOXA1. In the negative control group (PGL3-Basic), there was no significant difference in luciferase activity between cells transfected with pcDNA3.1(+) and pcDNA3.1( + )-TF (FOXA1). However, in the wild-type reporter group (PGL3-WT), co-transfection with FOXA1 significantly increased luciferase activity compared to the vector control, indicating that FOXA1 enhances SLC7A11 promoter activity. Mutation of either TFBS1 or TFBS2 (Mut1, Mut2) abolished this effect, suggesting these sites are essential for FOXA1-mediated transcriptional activation and that their disruption may cause transcriptional de-repression or off-target effects (Fig. 5C).

To further confirm the FOXA1-SLC7A11 interaction, CUT&Tag assays were performed in HEK293T cells. Following immunoprecipitation with a ChIP-grade anti-FOXA1 antibody, DNA fragments were assessed by agarose gel electrophoresis. No bands were detected in the control group (HEK293T) or the IgG control group, while distinct amplification bands appeared after 21 PCR cycles in the FOXA1-IP group (Fig. 5D). qPCR with primers targeting the SLC7A11 promoter showed a significant enrichment of this region in the FOXA1-IP group compared to controls, validating the specific interaction (Fig. 5E). In both PC-3 (Fig. 5F–J) and DU145 (Fig. 5K–O) cells, sequential transfection of SE-sgRNA-1, SE-sgRNA-2, and SE-sgRNA-3 led to a progressive decrease in FOXA1 mRNA and protein levels. Correspondingly, SLC7A11 expression was also significantly reduced, highlighting the co-expression relationship and regulatory axis between SEs, FOXA1, and SLC7A11. To further elucidate the critical role of the FOXA1 promoter, a specific CRISPR sgRNA targeting the FOXA1 promoter region (sgRNA-p) was designed as a positive control. Deletion of this promoter region mimicked the effects of SE-sgRNA transfection, resulting in a significant downregulation of both FOXA1 and SLC7A11 expression. These results confirm that FOXA1 directly regulates SLC7A11 as a transcription factor and that sequential disruption of FOXA1-associated SEs effectively silences this oncogenic axis.

To further investigate the downstream metabolic consequences of SLC7A11 regulation, we performed targeted metabolomics profiling in PC-3 and DU145cells following SLC7A11 knockout, covering 106 metabolites related to carbohydrate, amino acid, and nucleotide metabolism. Under FC-based screening, both cell lines exhibited pronounced metabolic reprogramming, with common differential metabolites mainly involved in amino acid metabolism (e.g., L-alanine, L-serine, L-asparagine), organic acid metabolism (e.g., malic acid, tartaric acid), carbohydrate metabolism intermediates (e.g., 6-phospho-D-gluconate, glucose-6-phosphate), and nucleotide metabolism (e.g., guanosine monophosphate, inosine monophosphate) (Supplementary Fig. 2A-B). When applying combined FC and VIP thresholds, the common differential metabolites retained in both cell lines were L-alanine, L-serine, and malic acid (Supplementary Fig. 2C-D). KEGG pathway enrichment analysis revealed significant enrichment of these metabolites in the tricarboxylic acid (TCA) cycle, alanine/aspartate/glutamate metabolism, and central carbon metabolism in both cell lines, along with notable alterations in carbohydrate and sulfur metabolism pathways (Supplementary Fig. 2E-F). Together with our previous observations on redox homeostasis and disulfidptosis, these results suggest that SLC7A11 not only prevents disulfidptosis by regulating cystine uptake and glutathione synthesis but also reshapes central energy metabolism networks. This metabolic reprogramming may render prostate cancer cells highly sensitive to perturbations in glucose metabolism, as explored in the subsequent experiments.

### Effects of glucose inhibition on prostate cancer progression

**Fig. 6:**
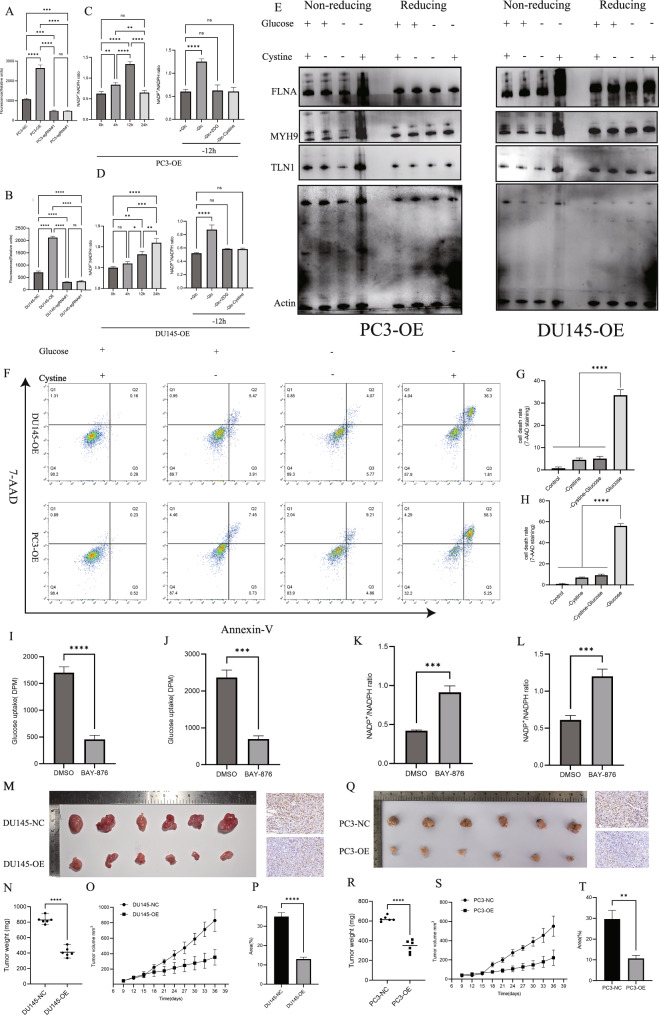
Effects of SLC7A11 on cellular metabolism. **A**, **B** Cysteine level in PC-3 and DU145 cells with varying SLC7A11 expression levels. **C**, **D** NADP⁺/NADPH ratio in SLC7A11-overexpressing (OE) cells under glucose-deprived conditions (−Glc) at different time points. **E** Reducing and non-reducing Western blot analysis showing the migration patterns of cytoskeletal proteins in SLC7A11-OE cells under different culture conditions. **F**–**H** Flow cytometry analysis of apoptosis in DU145-OE and PC-3-OE cells under glucose and/or cystine deprivation. **I**, **J** BAY-876 significantly inhibits glucose uptake in DU145 and PC-3 cells. **K**, **L** BAY-876 treatment leads to increased NADPH consumption, as reflected by elevated NADP⁺/NADPH ratios in DU145 and PC-3 cells. **M**–**O** In vivo, BAY-876 treatment results in significantly reduced tumor volume, weight, and growth rate in DU145-OE xenografts compared to DU145-NC controls. **P** Immunohistochemistry reveals a marked reduction in Ki67-positive area in DU145-OE tumors compared to DU145-NC. **Q**–**S** BAY-876 also significantly suppresses tumor volume, weight, and growth rate in PC-3-OE xenografts relative to PC-3-NC controls. **T** Immunohistochemical staining indicates decreased Ki67 expression in PC-3-OE tumors compared to PC-3-NC tumors.

Based on this, we further evaluated the effects of glucose restriction on prostate cancer progression. SLC7A11 primarily functions as a transporter of extracellular cystine into the cell, and the imported cystine is rapidly reduced to cysteine in the cytoplasm. In this study, overexpression of SLC7A11 significantly enhanced cysteine level in DU145 and PC-3 prostate cancer cells, whereas knockout led to a marked reduction in cysteine level (Fig. 6A, B). Under glucose-deprived conditions (-Glc), the NADP^+^/NADPH ratio gradually increased in DU145-OE cells, with a significant elevation observed at 12 h (Fig. 6C). In PC-3-OE cells, this elevation was detected as early as 4 h (Fig. 6D), suggesting that NADPH is rapidly consumed in SLC7A11-overexpressing cells when glucose is absent. After 12 hours of glucose deprivation, the NADP^+^/NADPH ratio was significantly higher than under complete medium conditions. Glucose supplementation (-Glc+Glc) markedly mitigated this increase, and 2-deoxyglucose (2-DG, -Glc+2DG) also reversed the elevation, though 2-DG cannot enter the pentose phosphate pathway (PPP) [10]. In both PC-3 and DU145 cell lines, glucose deprivation led to a pronounced reduction in G6P levels (Supplementary Fig. 2G-H). G6PD activity displayed cell line–specific alterations at early time points but declined at later stages in both cell lines (Supplementary Fig. 2I–J). These findings suggest that the primary driver of NADPH depletion is the diminished availability of PPP substrates under glucose-limiting conditions; in such a substrate-restricted state, even normal expression of PPP enzymes is unlikely to sustain pathway flux, and the subsequent reduction in G6PD activity may further exacerbate redox imbalance. In redox-sensitive Western blotting, no tailing of protein bands was observed under reducing conditions regardless of glucose or cystine status. However, under non-reducing conditions, glucose deprivation led to abnormal disulfide bond formation in cytoskeletal proteins such as FLNA, MYH9, TLN1, and Actin, resulting in band tailing. This effect was rescued by cystine deprivation (Fig. 6E). Notably, as shown in Supplementary Fig. 2K, under non-reducing conditions we consistently and reproducibly observed pronounced high-molecular-weight band tailing/aggregation of cytoskeletal proteins, including FLNA and Actin, upon glucose deprivation, with improved resolution further visualizing this disulfide bond–dependent phenotype, thereby reinforcing its specificity and robustness. Consistently, glucose deprivation significantly increased apoptosis in OE cells, while additional cystine deprivation reversed this effect (Fig. 6F–H), suggesting that cystine deprivation can restore redox balance by reducing NADPH consumption and suppressing apoptosis.

BAY-876, a selective glucose transporter 1 (GLUT1) inhibitor, was used to inhibit glucose uptake in complete medium. Previously, BAY-876 was shown to have IC_50_ values of 2.19 µM in DU145 and 0.70 µM in PC-3 cells. At these concentrations, BAY-876 significantly reduced glucose uptake (Fig. 6I–L) and increased the NADP^+^/NADPH ratio, implying that BAY-876 promotes NADPH depletion through interference with glucose metabolism. In vivo, prostate cancer cells were subcutaneously injected into nude mice. When tumor volumes reached 80 mm³, mice received intraperitoneal injections of BAY-876 solution (0.1 mL/10 g) every other day. In DU145 xenografts, tumors in the OE group exhibited smaller volume and mass than those in the NC group (Fig. 6M, N), with growth inhibition becoming apparent after day 21 (Fig. 6O). Immunohistochemistry showed significantly reduced ki67 expression in tumors from the OE group compared to the NC group (Fig. 6P), which contrasts with previous tumorigenesis experiments without treatment, suggesting that BAY-876 specifically suppresses SLC7A11-high tumors in vivo. Similarly, in PC-3 xenografts, the OE group exhibited significantly reduced tumor volume and weight compared to NC (Fig. 6Q, R), with differences emerging by day 15 (Fig. 6S). Immunohistochemical staining also showed lower ki67 expression in OE tumors (Fig. 6T). Furthermore, we performed immunohistochemical staining of 4-HNE and cleaved caspase-3 on subcutaneous tumor tissues (Supplementary Fig. 2L-N). No significant differences were observed among groups, suggesting that neither ferroptosis nor apoptosis was involved. These findings are consistent with the reported features of disulfidptosis, which lacks canonical apoptotic and ferroptotic markers. These results demonstrate that BAY-876 effectively inhibits the growth of SLC7A11-high prostate tumors in vivo by disrupting glucose uptake and promoting oxidative stress.

### Role of FOXA1-associated super-enhancers in regulating disulfidoptosis

**Fig. 7:**
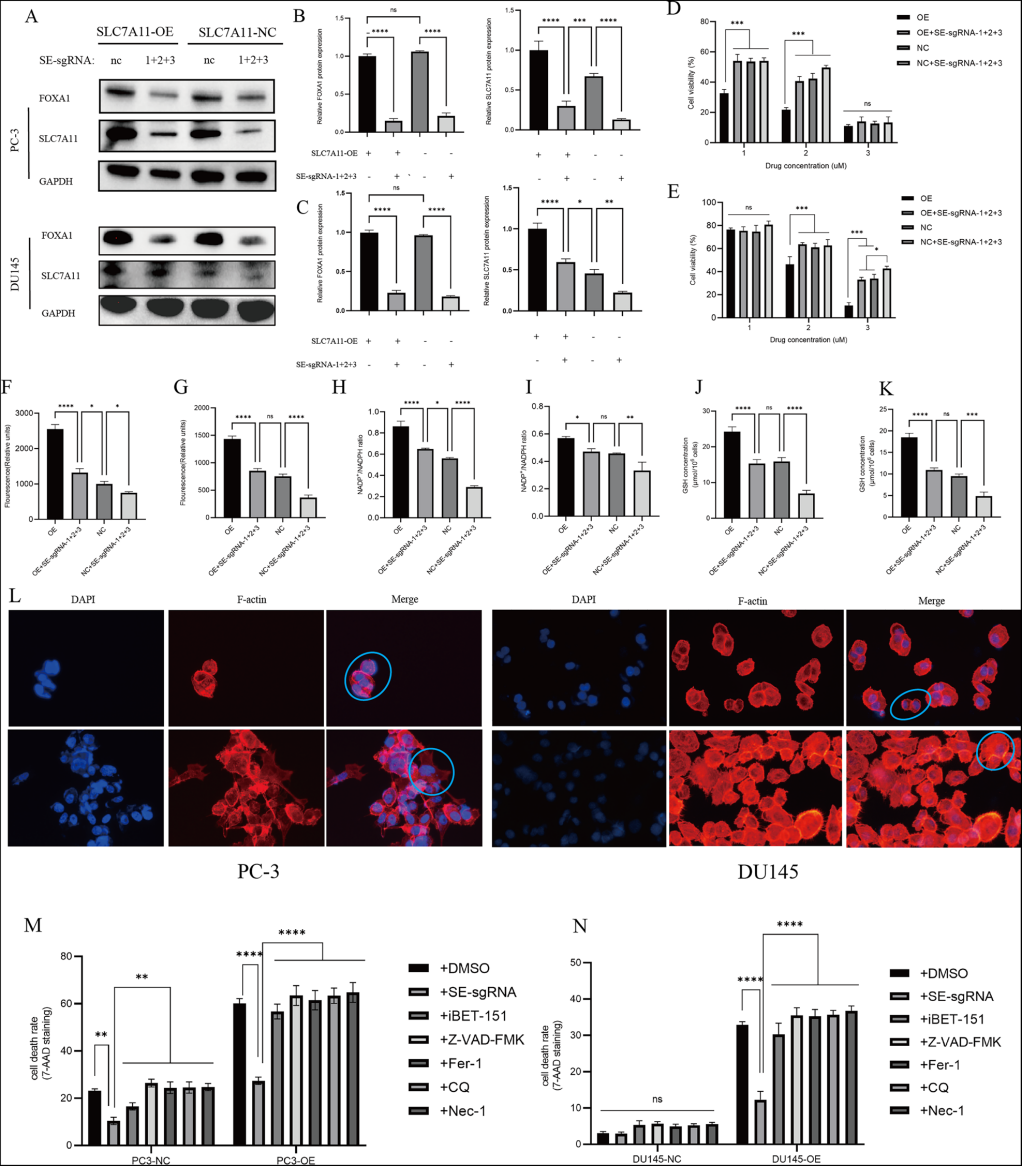
Effects of FOXA1-associated super-enhancers on metabolic regulation in PC-3 and DU145 prostate cancer cells. **A**–**C** Changes in FOXA1 and SLC7A11 expression following FOXA1-SEs deletion in PC-3 and DU145 cells. **D**, **E** Impact of SE-sgRNA-mediated FOXA1-SEs deletion on BAY-876 sensitivity in PC-3 and DU145 cells. **F**, **G** Effects of SE-sgRNA on cysteine level in PC-3 and DU145 cells. **H**, **I** Effects of SE-sgRNA on NADP⁺/NADPH ratios in PC-3 and DU145 cells (1 µM). **J**, **K** SE-sgRNA-mediated alterations in intracellular GSH levels in PC-3 and DU145 cells. **L** F-actin and DAPI staining showing FOXA1-SEs influence on cytoskeletal morphology under glucose deprivation. **M**, **N** Apoptosis rates in PC-3 and DU145 cells under different treatments, including SE-sgRNA and various regulated cell death inhibitors.

To investigate the regulatory effect of FOXA1-associated super-enhancers (SEs) on SLC7A11, sequential transfection of SE-sgRNA-1, -2, and -3 was performed in both SLC7A11-overexpressing (OE) and control (NC) DU145 and PC-3 cells, thereby establishing stable FOXA1-SE-inhibited cell lines. Quantitative analysis showed that SE-sgRNA significantly downregulated both FOXA1 mRNA and protein levels. Consequently, SLC7A11 expression was also reduced, particularly in the OE group, where high SLC7A11 levels were partially reversed by SE-sgRNA (Fig. 7A–C). These findings suggest that FOXA1-SEs not only directly control FOXA1 expression but also indirectly modulate downstream SLC7A11 levels.

In PC-3 cells treated with increasing concentrations of BAY-876 (1, 2, 3 µM), SLC7A11 overexpression sensitized cells to glucose deprivation, resulting in decreased cell viability. However, in the OE + SE-sgRNA group, viability was partially restored, indicating that deletion of SEs reduces the drug sensitivity caused by SLC7A11 overexpression (Fig. 7D). Notably, at 2 µM BAY-876, the NC + SE-sgRNA group exhibited the highest viability, supporting the hypothesis that SEs influence cellular tolerance to glucose metabolism stress. In DU145 cells, no significant differences in viability were observed among groups at 1 µM BAY-876. However, at 2 µM and 3 µM, OE cells showed a pronounced decrease in viability, while NC + SE-sgRNA cells maintained higher survival rates (Fig. 7E).

Cysteine uptake assays revealed that SLC7A11 overexpression increased cysteine level in both cell lines. SE-sgRNA transfection reduced cysteine level in OE cells. In PC-3, the OE + SE-sgRNA group still showed higher level than NC, while in DU145, OE + SE-sgRNA and NC groups showed no significant difference (Fig. 7F, G). Similarly, the NADP⁺/NADPH ratio increased in OE cells and was partially reversed by SE-sgRNA treatment. This reversal was more pronounced in PC-3 (Fig. 7H) than in DU145 (Fig. 7I) (1 µM). Glutathione (GSH) concentrations were significantly elevated in OE cells in both lines. SE-sgRNA treatment reduced GSH levels in both NC and OE groups, eliminating the differences in GSH concentrations between groups (Fig. 7J, K). These results indicate that deletion of FOXA1-SEs disrupts SLC7A11-driven redox balance by lowering cysetine uptake and reducing NADPH and GSH levels. Under glucose-deprived conditions, DAPI and F-actin staining revealed collapsed cytoskeletal structures in OE cells, with compact, rounded morphologies (blue boxes). In contrast, OE + SE-sgRNA cells retained intact cytoskeletal architecture (Fig. 7L), further supporting the role of FOXA1-SEs in mediating disulfidoptosis.

In the basal culture medium, NADP⁺/NADPH levels increased with elevated SLC7A11 expression in PC-3 (Supplementary Fig. 3A) and DU145 cells (Supplementary Fig. 3B). Following H₂O₂ treatment, oxidative stress enhanced the consumption rate of NADPH in all groups, with the OE group showing the most significant increase in the NADP⁺/NADPH ratio. Meanwhile, under oxidative stress, the rapid depletion of NADPH in the OE group failed to meet the demand for GSH regeneration, resulting in a rapid exhaustion of GSH and an increase in GSSG, thereby elevating the GSSG/GSH ratio. In the OE + SE-sgRNA group, due to reduced NADPH consumption, GSH levels were able to recover. As a result, compared with the OE group, the GSSG/GSH ratio was suppressed, although still significantly higher than in the NC or NC + SE-sgRNA groups (Supplementary Fig. 3C, D). These findings suggest that under oxidative stress conditions, high levels of SLC7A11 can lead to an imbalance in the antioxidant system, consistent with previous research. This process can be rescued by FOXA1-SEs, which help maintain the balance of NADPH and GSH. In summary, these results indicate that the loss of FOXA1-SEs may affect the levels of redox metabolites and participate in the regulation of tumor metabolism.

**Fig. 8:**
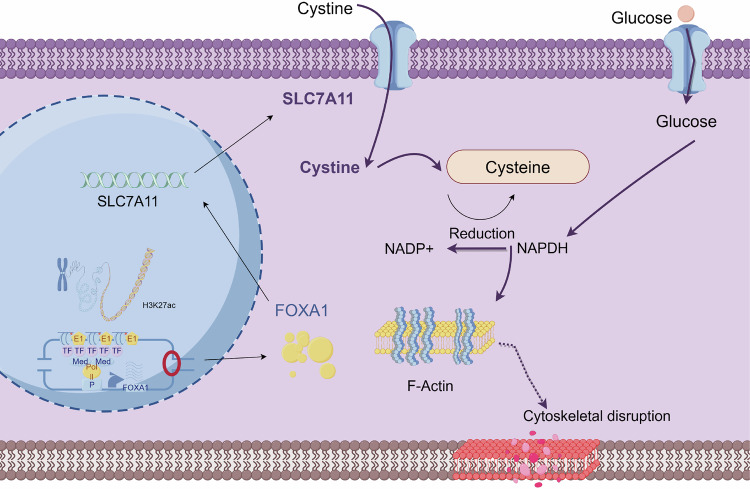
The molecular regulatory mechanism of SEs-FOXA1-SLC7A11 in disulfidptosis (By Figdraw).

To explore the involvement of FOXA1-SEs in regulated cell death, a panel of cell death inhibitors was applied, including the pan-apoptosis inhibitor Z-VAD-FMK (10 μM), ferroptosis inhibitor Ferrostatin-1 (2 μM), autophagy inhibitor Chloroquine (60 ng/mL), and necroptosis inhibitor Necrostatin-1 (10 μM). In DU145-NC cells, neither SE-sgRNA nor any inhibitor significantly altered late apoptosis rates. However, in DU145-OE cells, SE-sgRNA treatment markedly reduced late apoptosis, whereas the other inhibitors failed to rescue disulfidoptosis (Fig. 7M). Interestingly, iBET-151, a SE inhibitor, mildly reduced apoptosis rates in both OE and NC groups, but did not reach statistical significance compared to DMSO, likely due to its inherent cytotoxic effects. In PC-3 cells, SE-sgRNA and iBET-151 significantly reduced the high apoptosis rates observed in the NC group, while other inhibitors were ineffective (Fig. 7N). In the OE group, SE-sgRNA consistently attenuated late apoptosis, whereas iBET-151 and other inhibitors showed no rescue effects (Fig. 7O). Collectively, these findings demonstrate that deletion of FOXA1-SEs significantly inhibits SLC7A11-induced disulfidoptosis in prostate cancer cells (Supplementary Fig. 3E, F). Standard inhibitors of apoptosis, ferroptosis, autophagy, and necroptosis were unable to prevent this unique form of cell death, highlighting the specificity of SEs in this pathway. This study identifies FOXA1-SEs as key regulators of disulfidoptosis and offers new therapeutic insight into metabolic intervention strategies in prostate cancer. These results suggest that FOXA1-SEs play a key role in regulating disulfidptosis. Figure 8 shows the molecular regulatory mechanism of SEs-FOXA1-SLC7A11 in disulfidptosis.

To further validate this regulatory relationship, we performed a functionally equivalent rescue experiment in DU145 and PC-3 cells with four groups: NC, OE, NC + SE-sgRNA, and OE + SE-sgRNA. At the molecular level, Western blot analysis showed that FOXA1 overexpression markedly increased FOXA1 and SLC7A11 protein levels, whereas SE inhibition in the OE background significantly reduced their expression to NC levels; SE inhibition in the NC background also markedly decreased FOXA1 and SLC7A11 expression (Supplementary Fig. 4A). At the functional level, Annexin V/PI flow cytometry revealed that FOXA1 overexpression reduced cell death under glucose deprivation, whereas SE inhibition in the OE background restored the death rate to near NC levels, and the NC + SE-sgRNA group showed the lowest death rate with normal morphology (Supplementary Fig. 4F–H). Furthermore, F-actin immunofluorescence staining demonstrated that FOXA1 overexpression re-induced cytoskeletal collapse—a morphological hallmark of disulfidptosis—which was absent in both OE + SE and NC + SE groups (Supplementary Fig. 4I). These data indicate that the FOXA1-associated SE plays a pivotal role in regulating SLC7A11, primarily by modulating FOXA1 expression.

## Discussion

SLC7A11, a member of the amino acid transporter family, is primarily responsible for mediating cystine import into the cell. It has been shown to play crucial roles in multiple cancers. In lung cancer, for instance, SOX2 can activate SLC7A11 expression, enhancing resistance to ferroptosis and increasing tumor aggressiveness and drug resistance [21]. In gastric cancer, SLC7A11-AS1 inhibits SLC7A11 expression, thereby influencing cisplatin resistance and tumor progression [22]. In breast cancer, decreased cystine levels in the tumor microenvironment remodel the expression of KCTD10 and USP18, regulating SLC7A11 stability and subsequently cystine metabolism [23]. In prostate cancer, SLC7A11 has been shown to sensitize tumors to ferroptosis inducers such as erastin and RSL3, which significantly inhibit tumor growth, especially when combined with second-generation antiandrogens like enzalutamide or abiraterone [24]. Moreover, SLC7A11 is highly expressed in therapy-resistant tumors. In docetaxel-resistant cells, both GPX4 and SLC7A11 are upregulated, while erastin and RSL3 can restore chemosensitivity [25]. Interference with circDUSP22 increases ferroptosis by downregulating SLC7A11, and SLC7A11 overexpression reverses this effect, further validating its pro-proliferative role in PCa [26].

In this study, we constructed a diagnostic model using transcriptomic data from 837 prostate cancer samples and identified SLC7A11 as a key factor in tumor progression. Expression analyses revealed its significant upregulation in prostate tumors, especially in high Gleason score samples, suggesting a correlation with tumor aggressiveness. Both in vitro and in vivo experiments confirmed SLC7A11’s functions in regulating cell survival, migration, morphology, and tumor growth. Notably, we observed a new function of SLC7A11 in modulating cytoskeletal remodeling under glucose-deprived conditions in PCa, implicating it in the induction of disulfidoptosis during tumor development. Metabolic adaptation is a hallmark of cancer, allowing tumor cells to survive in hypoxic, nutrient-deprived microenvironments. SLC7A11 overexpression has been linked to redox balance regulation [27]. In poorly perfused tumor cores, limited glucose and oxygen necessitate metabolic reprogramming for survival. SLC7A11 enables cystine and GSH uptake, maintaining redox homeostasis. However, GSH reduction is NADPH-dependent, and NADPH is primarily generated via the pentose phosphate pathway (PPP), a glucose-driven process. Thus, SLC7A11-high cells become highly glucose-dependent. This metabolic vulnerability aligns with disulfidoptosis, offering a unique therapeutic window in late-stage tumors. Our study demonstrates that BAY-876, a GLUT1 inhibitor, effectively induces disulfidoptosis by limiting glucose uptake and disrupting NADPH regeneration, significantly reducing tumor growth in SLC7A11-high xenograft models. Previous studies support BAY-876’s utility in cancer therapy. For instance, Xijiao Ren et al. [28] developed a DNA-PAE@BAY-876 nanodelivery platform to reprogram metabolism and improve immune responses in triple-negative breast cancer. Yang Luo et al. [29] achieved simultaneous inhibition of glycolysis, glucose uptake, and autophagy using BAY-876, 2-DG, and chloroquine-loaded nanoclusters. Jia-Wei Wang et al. [30] co-delivered paclitaxel and BAY-876 using HSA nanoparticles to activate AMPK signaling and enhance chemotherapy. Similarly, James H. Joly et al. [31] reported that co-targeting glucose transport and GSH synthesis produced synthetic lethality in SLC7A11-high tumors. Despite these advances, BAY-876 remains underexplored in prostate cancer. With ongoing innovations in nanomedicine and drug delivery, BAY-876 or its derivatives may serve as promising agents for targeting SLC7A11-driven metabolic vulnerabilities in advanced PCa.

Our study also identified FOXA1 as a critical transcription factor regulating SLC7A11. Multi-omics and CUT&Tag analyses confirmed FOXA1’s binding to the SLC7A11 promoter and its transcriptional activation. Interestingly, FOXA1 itself was shown to be regulated by SEs. In the context of CRPC, SE reprogramming is recognized as a key mechanism. Regulatory proteins such as BRD4 and MED1 aggregate via liquid–liquid phase separation at SE loci, facilitating transcriptional complex assembly and oncogene activation. For example, darolutamide blocks AR binding to enhancer elements, thereby inhibiting androgen-driven gene expression and tumor proliferation [32]. Similarly, ONECUT2 drives lineage plasticity by altering chromatin accessibility and SE activation, promoting NEPC features and AR-independent pathways [33]. In this study, we performed epigenomic profiling across multiple prostate cancer cell lines and identified high-density H3K27ac regions on chromosome 14. Through SE-gene mapping and CRISPR-Cas9 deletion, FOXA1 was confirmed as an SE target gene. Sequential deletion of SE elements progressively suppressed FOXA1 expression. This study is the first to define the FOXA1 SE region in PCa and validate its function. SE inhibition also led to reduced SLC7A11 expression, decreased antioxidant capacity, and increased cell death. FOXA1 functions both as a transcriptional regulatory factor and a driver activated by SEs, playing diverse roles in prostate cancer. Previous studies on FOXA1 have primarily focused on how FOXA1 mutations regulate AR activity. However, this study does not involve changes in the Forkhead domain and mainly investigates the impact of basal FOXA1 levels on tumor biology, given that PC-3 and DU145 are AR-negative cell lines. In AR-positive cells, upregulation of FOXA1 enhances the viability of C4-2 cells [34] and is considered a key factor in driving the life cycle and proliferation of LNCaP and 22RV1 cells [35]. However, a study by Leiming Wang et al. found that overexpression of FOXA1 in LNCaP cells actually inhibited invasion [36]. This study also showed that BET inhibitors such as JQ-1 and iBET-151 could reduce the viability of AR-positive prostate cancer cells, which differs from the findings of Sakshi Goel et al., who reported that JQ-1 suppressed the viability of VCaP and 22RV1 cells [37]. Our research also found that iBET-151 can accelerate apoptosis, and such discrepancies may be related to different drug concentrations used in experiments. In AR-negative cells, research by Josefine Gerhardt et al. [38] found that inhibiting FOXA1 in PC-3 cells significantly reduced DNA synthesis rates and cell viability. Rehanna Mansor [39] observed that silencing FOXA1 reduced migration and invasion in DU145 cells. However, other studies have found that increased FOXA1 expression can suppress the migration and invasion of PC-3 and DU145 cells [40, 41]. These results suggest that FOXA1 can mediate cancer cell invasion and metastasis independently of the AR signaling pathway. Possible reasons for inconsistent results may include the diversity of FOXA1 mutants during cell culture, differences in metabolic components in the culture environment, or variability in intrinsic cellular states. A potential explanation offered by this study is that FOXA1 expression has a bidirectional regulatory effect on cellular oxidative stress tolerance, exhibiting a characteristic “double-edged sword” behavior. Under high SLC7A11 expression, moderate inhibition of FOXA1 may enhance tumor cell survival, while at basal levels, it appears to have a suppressive effect. In terms of metabolism, the FOXA1-SEs-SLC7A11 pathway may connect redox metabolism with programmed cell death, particularly disulfidptosis. In addition to regulating FOXA1, super-enhancers may also be involved in processes such as glucose metabolism, lipid metabolism, and amino acid metabolism, thereby driving cancer cell adaptation to extreme microenvironments. Our findings offer new insights into the role of SEs in metabolic reprogramming.

Despite these findings, our study has limitations. First, co-expression patterns of FOXA1 and SLC7A11 were not validated in clinical samples. Second, SE–gene interactions were not directly tested using chromatin conformation assays (e.g., Hi-C, ChIA-PET, 3 C/qPCR); SE identification relied on combined peak analyses without base-pair resolution, which may obscure enhancer boundaries and affect functional interpretation. Finally, the role of SEs in disulfidoptosis requires more direct evidence, and orthotopic models were not used to mimic metastatic processes.

## Conclusion

Under physiological conditions, SLC7A11 promotes the proliferation of prostate cancer cells (PC-3 and DU145) both in vitro and in vivo. Under glucose-deprived conditions, high SLC7A11 expression leads to cytoskeletal collapse and triggers disulfidoptosis, a newly characterized form of cell death. The glucose transporter inhibitor BAY-876 significantly suppresses tumor growth in SLC7A11-high prostate cancer models, highlighting a potential therapeutic vulnerability. Notably, the super-enhancer regions regulating FOXA1 were identified on human chromosome 14 (chr14:37583488–37589585). These enhancers exhibit a synergistic effect on FOXA1 expression. As a transcription factor, FOXA1 directly binds to the SLC7A11 promoter, and its super-enhancers modulate disulfidoptosis via SLC7A11. Together, these findings reveal a FOXA1–SE–SLC7A11 regulatory axis that connects transcriptional control with redox-dependent cell death in prostate cancer.

## Supplementary information

Supplementary Figure 1

Supplementary Figure 2

Supplementary Figure 3

Supplementary Figure 4

supplementary legands

Supplementary File 1

uncroped gels

## Data Availability

The data that support the findings of this study are available from the corresponding author upon reasonable request.
