# Supplementary figures and images for: Super-enhancers mediates SLC7A11 via FOXA1 to regulate disulfidptosis in prostate cancer
Source: Cell Death Dis. 2025 Dec 3;17(1):63. doi: 10.1038/s41419-025-08227-2 (PMC12827458; doi:10.1038/s41419-025-08227-2)

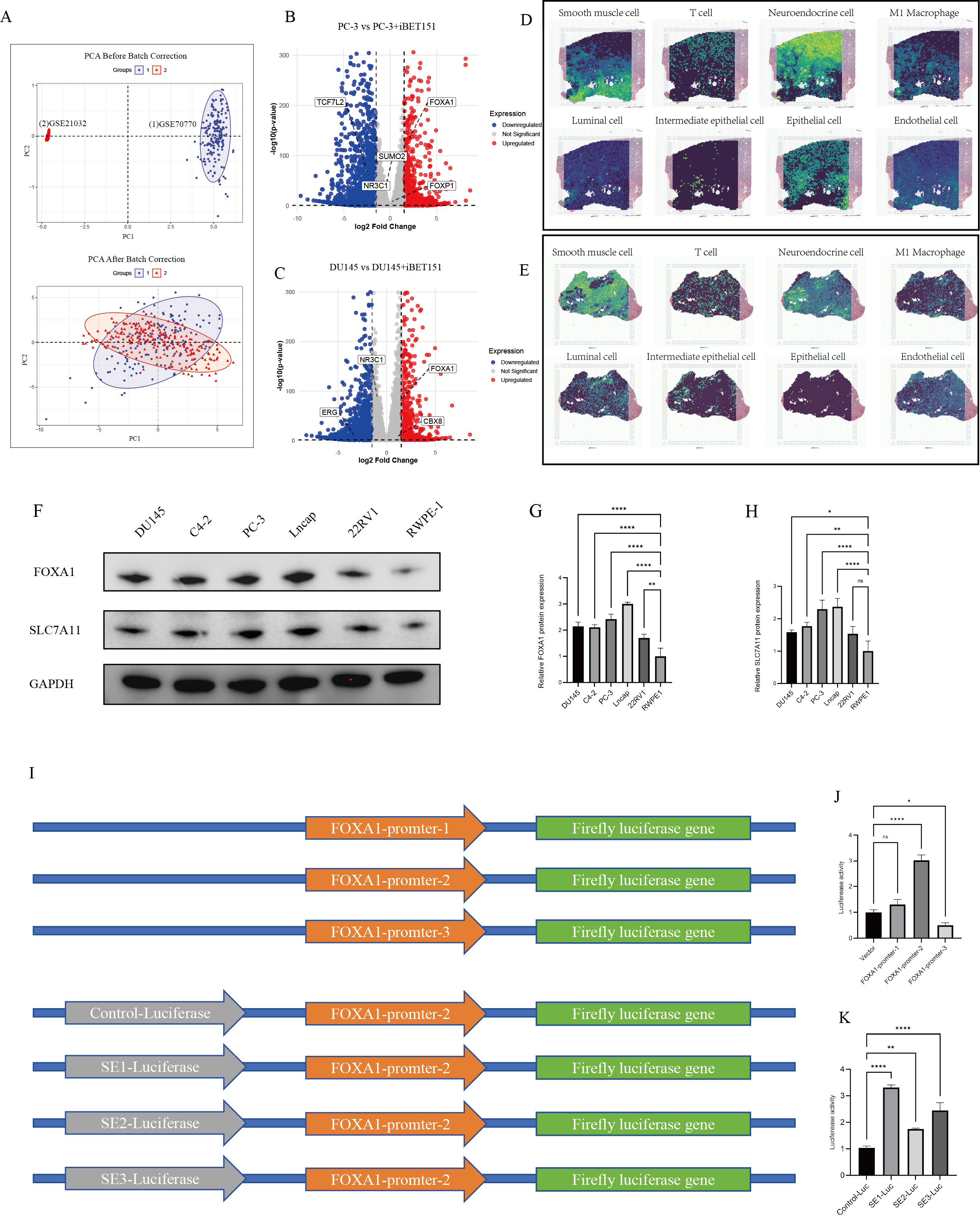

Supplement: Supplementary file 1 — Supplementary Figure 1 [file 41419_2025_8227_MOESM1_ESM.png]

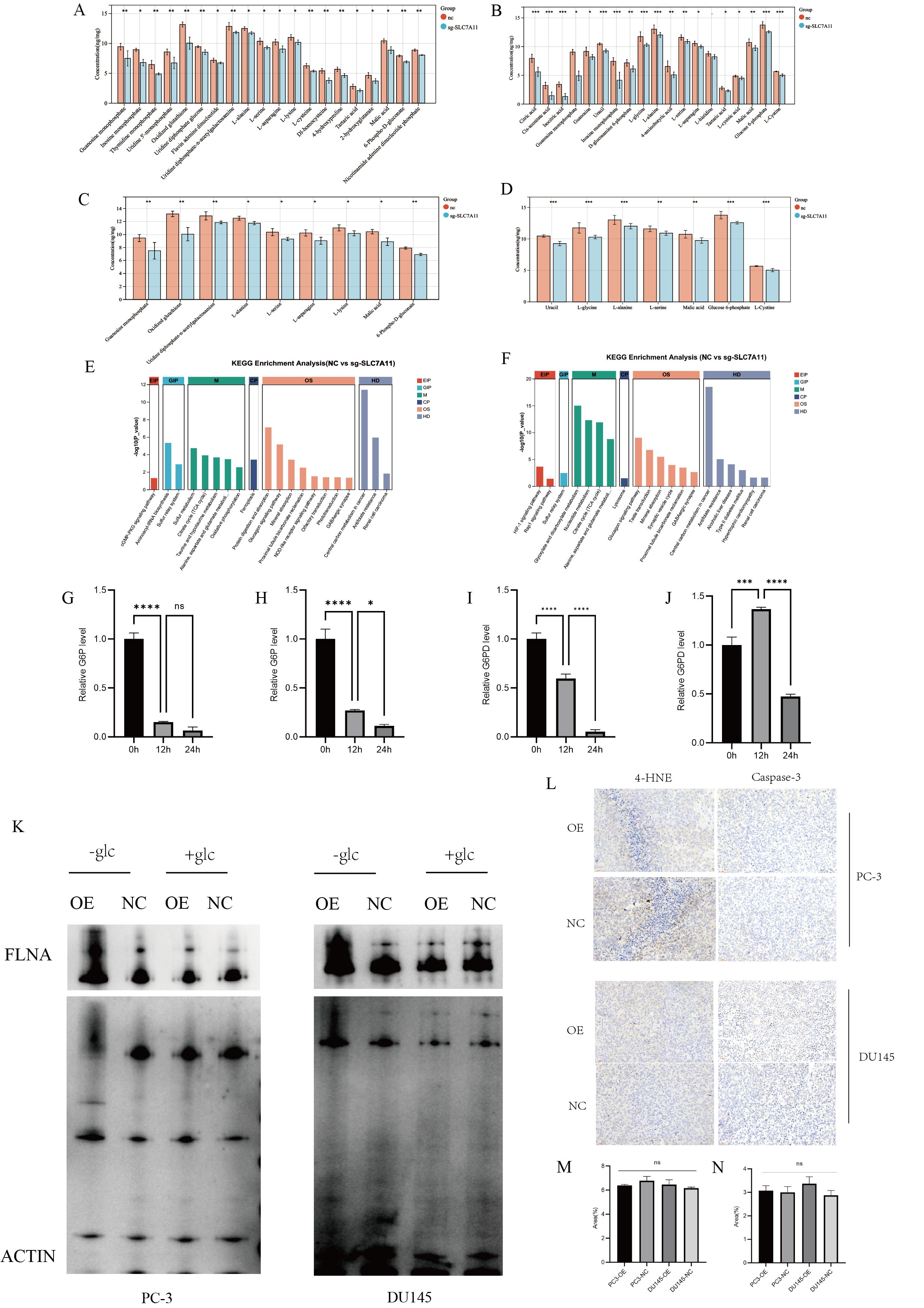

Supplement: Supplementary file 2 — Supplementary Figure 2 [file 41419_2025_8227_MOESM2_ESM.png]

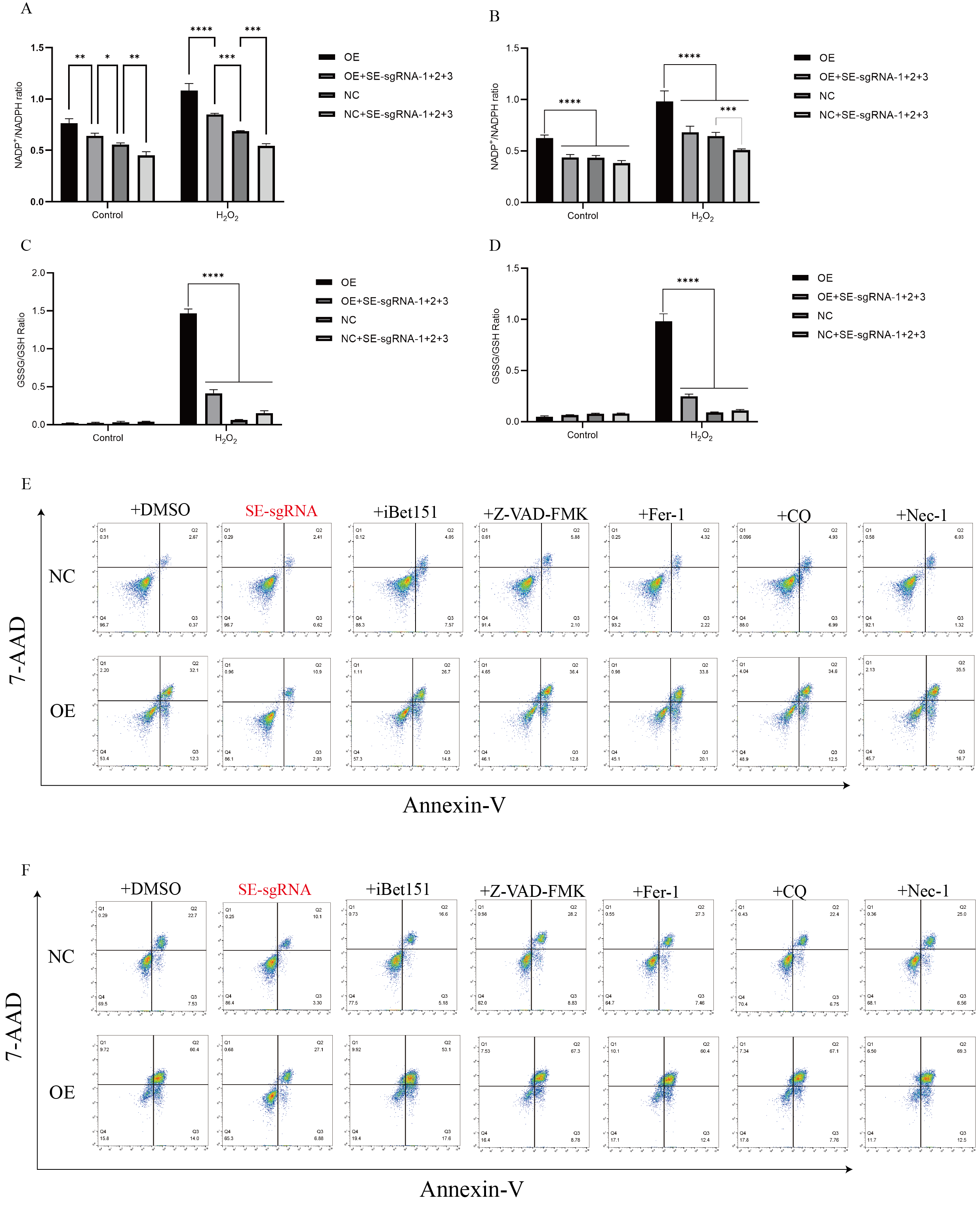

Supplement: Supplementary file 3 — Supplementary Figure 3 [file 41419_2025_8227_MOESM3_ESM.png]

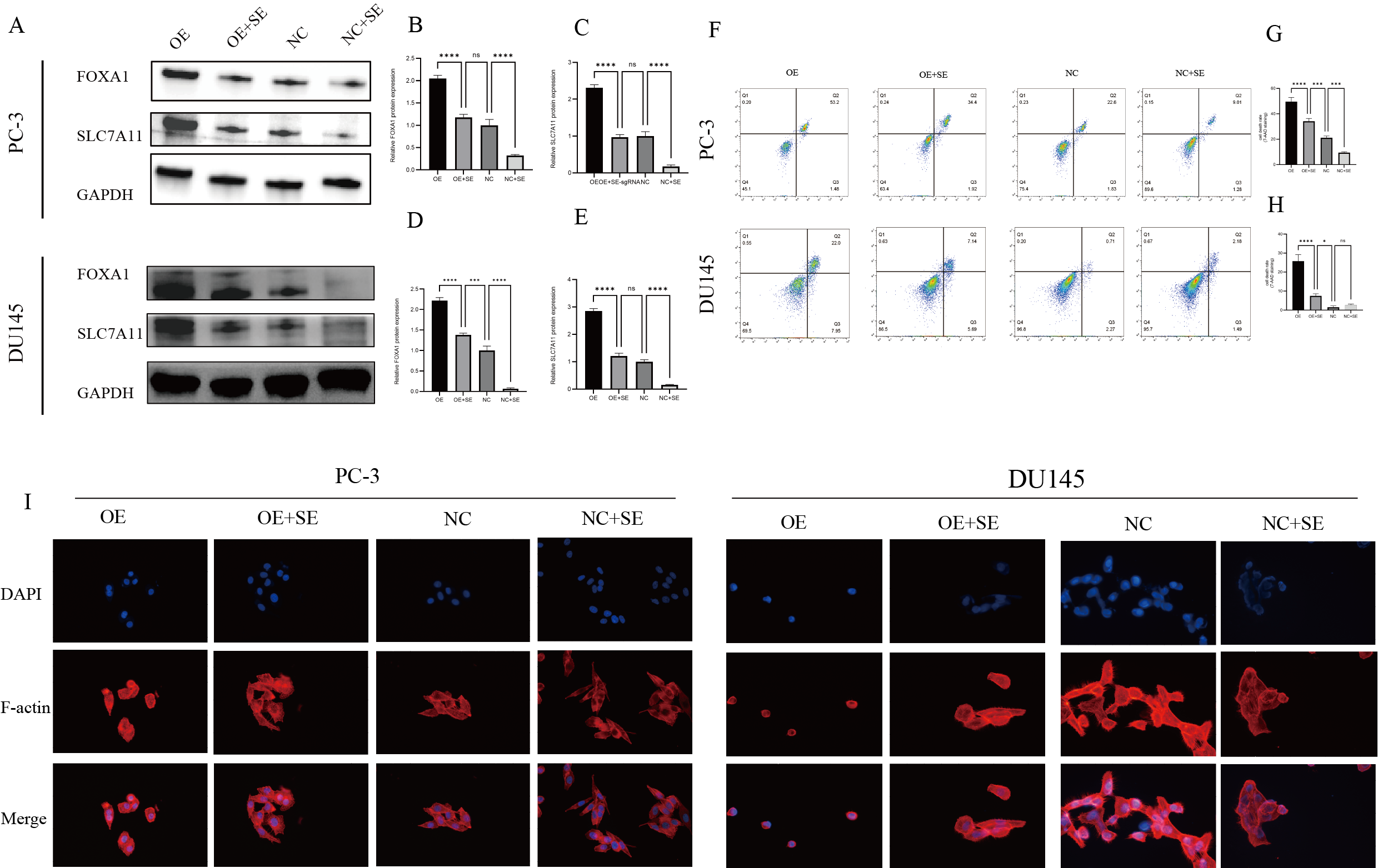

Supplement: Supplementary file 4 — Supplementary Figure 4 [file 41419_2025_8227_MOESM4_ESM.png]

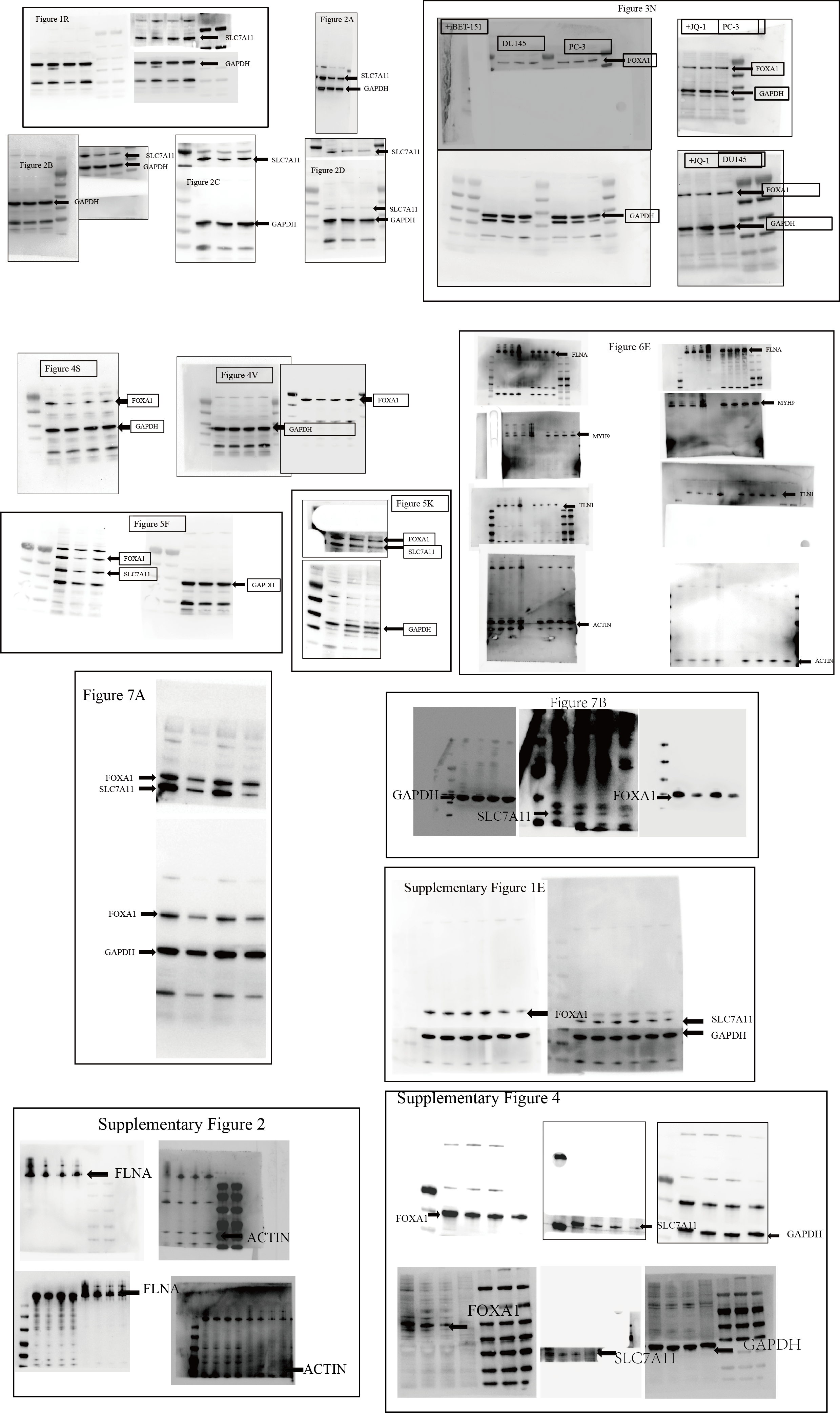

Supplement: Supplementary file 7 — uncroped gels [file 41419_2025_8227_MOESM7_ESM.png]
