# Supplementary material for: Super-enhancers mediates SLC7A11 via FOXA1 to regulate disulfidptosis in prostate cancer
Source: Cell Death Dis. 2025 Dec 3;17(1):63. doi: 10.1038/s41419-025-08227-2 (PMC12827458; doi:10.1038/s41419-025-08227-2)
Supplement: Supplementary file 5 — supplementary legands [file 41419_2025_8227_MOESM5_ESM.doc]

Supplementary Figure 1.(A) PCA plots of the GEO datasets (GSE70770 and GSE21032) before and after batch effect correction using the ComBat method. (B, C) Volcano plots from transcriptome sequencing. (D, E) Distribution of cell clusters in prostate cancer and normal prostate tissue. (F-H) Protein expression levels of FOXA1 and SLC7A11 in different cell lines (DU145, C4-2, PC-3, LNCaP, 22RV1, and RWPE-1). (I) Schematic diagram of FOXA1 promoter and SEs luciferase reporter constructs. The FOXA1 promoter fragments (FOXA1-promoter-1 chr14:37595254-37595625; FOXA1-promoter-2 chr14:37595660-37596192; FOXA1-promoter-3 chr14:37596224-37596633) were cloned into the PGL3-basic vector. The control DNA (control-luc: chr14: 37581627-37582333) and SE segments (SE-1-luc: chr14: 37583559-37585537; SE-2-luc: chr14: 37585874-37586620;SE3-luc: chr14: 37587283-37589522) were further inserted the FOXA1-promoter-2 constructs. (J,K) Dual luciferase reporter assays examined the core SE activity 24 h post-transfection in 293T cells. Firefly luciferase activity was measured and normalized to renilla luciferase, and was presented as a ratio relative to activity of the control vector.

Supplementary Figure 2. (A-B) Bar plots showing differentially abundant metabolites between SLC7A11 knockout (KO) and negative control (NC) groups in PC-3 (A) and DU145 (B) cells under FC > 1, p < 0.05 criteria; (C-D) Bar plots showing differential metabolites under combined FC > 1, p < 0.05, and VIP ≥ 1 thresholds in PC-3 (C) and DU145 (D) cells; (E-F) KEGG pathway enrichment analysis of differential metabolites in PC-3 (E) and DU145 (F) cells, highlighting significant enrichment in the TCA cycle, alanine/aspartate/glutamate metabolism, and central carbon metabolism. G6P levels (G, H) and G6PD activity (I, J) in PC-3 and DU145 cells after 0, 12, and 24 h of glucose deprivation. (K)Glucose deprivation induces band tailing of FLNA and ACTIN under non-reducing conditions in OE cells but not in NC controls. (L)Immunohistochemical analysis of cleaved caspase-3 and 4-HNE in subcutaneous tumor tissues from NC and OE groups. (M,N)No significant difference was observed between groups, as shown in representative images and quantification plots .

Supplementary Figure 3. (A-D) Regulatory effects of SEs on NADP^+^/NADPH and GSSG/GSH in PC-3 and DU145 cells;(E) Apoptosis rate of DU145 in each treatment group. (F) Apoptosis rate of PC-3 in each treatment group.

Supplementary Figure 4. (A) Western blot showing FOXA1 overexpression (OE) increased FOXA1 and SLC7A11 levels in DU145 and PC-3 cells, while SE inhibition (OE+SE-sgRNA) reduced them to near control (NC) levels. (F-H) Annexin V/PI flow cytometry showing OE reduced cell death under glucose deprivation, whereas SE inhibition restored death rates to NC levels. (I) F-actin staining showing cytoskeletal collapse in OE cells under glucose deprivation, which was absent after SE inhibition.
